# Mineral nutrition for *Cannabis sativa* in the vegetative stage using response surface analysis

**DOI:** 10.3389/fpls.2024.1501484

**Published:** 2024-12-03

**Authors:** Patrick Yawo Kpai, Oluwafemi Adaramola, Philip Wiredu Addo, Sarah MacPherson, Mark Lefsrud

**Affiliations:** Department of Bioresource Engineering, McGill University, Montreal, QC, Canada

**Keywords:** Hemp, medical cannabis, mineral requirements, nutrient use efficiency, response surface analysis, vegetative stage

## Abstract

Cannabis cultivated for medical and adult use is a high-value horticultural crop in North America; however, we lack information on its optimal mineral nutrition due to previous legal restrictions. This study evaluated the mineral requirements of nitrogen (N), phosphorus (P), and potassium (K) for cannabis in the vegetative stage using response surface analysis. Plants were cultivated in a hydroponic system with various nutrient solution treatments (mg L^-1^) of N (132.7, 160, 200, 240, and 267.3), P (9.6, 30, 60, 90, and 110.5), and K (20.8, 60, 117.5, 175, and 214.2) according to a central composite design. Nutrient interactions (N × K, K × P, and N × P × K) had a significant effect on the vegetative growth of the cannabis plants. N × K interaction had a significant effect on leaf mass and stem mass. K × P interaction had a significant effect on dry root mass, leaf mass, stem mass, leaf area, specific leaf area, and chlorophyll a and b contents. N × P × K interaction had a significant effect on root mass, leaf mass, stem mass, stem diameter, leaf area, and chlorophyll a and b contents. The optimum concentrations of total nitrogen, P, K, calcium, and sulfur in the cannabis leaves were 0.54, 0.073, 0.27, 0.56, and 0.38 mg g^-1^, respectively. An increase in P and K concentrations decreased the magnesium concentration in the leaves, but it was unaffected by the increase in N concentration. The recommended primary macronutrients for cannabis plants in the vegetative stage based on the maximum desirability and nutrient use efficiencies were 160–200 mg L^-1^ N, 30 mg L^-1^ P, and 60 mg L^-1^ K. These findings can offer valuable insight and guidance to growers regarding the mineral requirements for cannabis during the vegetative stage.

## 1 Introduction

Sustainability is a widely used concept in various areas of our lives, especially in agriculture due to the environmental effects of certain crop production techniques (Atkinson and McKinlay, 1997; Hanson et al., 2007; Spicka et al., 2019). Sustainable agriculture can be described as managing agricultural systems to maintain a proper balance of biological diversity, productivity, and regenerative capacity, meeting current and future needs while avoiding harm to other ecosystems (Lewandowski et al., 1999). This approach entails managing plant diseases and nutrients to enhance both product yield and quality (Camprubí et al., 2007).

Effective fertilizer strategies and nutrient solutions in hydroponics are crucial for advancing sustainable agriculture, boosting yield and quality, and managing pests and diseases (Dordas, 2008; Gupta et al., 2017). Mineral nutrients stimulate enzymes that are key to producing defense metabolites and have a direct impact on plant health (Datnoff et al., 2007). Adequate and well-balanced plant nutrition serves as the primary defense, as mineral elements play a direct role in safeguarding plants (Tripathi et al., 2022). Mineral nutrients and primary macronutrients, in particular, are among the main environmental factors that have an impact on plant development, physiology, and metabolism (Lea and Morot-Gaudry, 2001; Saloner and Bernstein, 2022). Nitrogen (N), phosphorus (P), and potassium (K) are the three primary macronutrients vital for various aspects of plant metabolism (Gorelick et al., 2019).

In many agricultural regions of the world, nutrient runoff is a problem due to excessive nutrient application, particularly N and P, which can cause water bodies to become eutrophic (Schindler et al., 2016). The disposal of waste greenhouse nutrient solutions, including that from cannabis production facilities, is regulated by law in Canada and comes at a significant financial burden to growers (Bevan et al., 2021). To better balance the supply and demand of nutrients to enhance output while minimizing nutrient loss and the associated negative effects on the environment, it is imperative to understand the mineral nutrient requirements of the cannabis (*Cannabis sativa*) plant.

Few studies have investigated the response of cannabis to N, P, and K during the vegetative stage, and the examination of cannabis’ response to mineral nutrients remains largely unexplored (Bevan et al., 2021). Previous cannabis research that focused on nutrients have either used varying concentrations of NPK in fixed ratios (Caplan et al., 2017a) or examined varying concentrations of one mineral nutrient while keeping the others unchanged (Saloner et al., 2019; Saloner and Bernstein, 2021; Shiponi and Bernstein, 2021). This could significantly affect the recommendations for optimal application rates, as neither approach can assess nutrient interaction effects (Bevan et al., 2021).

Response surface methodology (RSM) offers a different approach to experimentation with the capability of optimizing multiple factors concurrently over a wide range of levels with fewer experimental units when compared to other experimental designs (Myers et al., 2016). Some researchers have approached the optimization of nutrient solutions as a “mixture system”, which is a form of multifactor optimization similar to RSM (De Rijck and Schrevens, 1998). An experimental design that uses a mixture system only optimizes the nutrient composition of the solution without altering the overall nutrient concentration, as it keeps the overall supply of nutrients consistent (Bevan et al., 2021). RSM overcomes this limitation by enabling the optimization of both the composition of the nutrient solution and the concentrations of its individual components (Bevan et al., 2021).

This study aimed to evaluate the mineral requirements and interactions of NPK for the vegetative stage of cannabis in a hydroponic system using RSM. The results of this study may improve our understanding of cannabis’ NPK requirements in the vegetative stage, and the findings highlight the effects of nutrient interaction on the vegetative properties of *C. sativa*.

## 2 Materials and methods

### 2.1 Plant and growing conditions

**Figure 1:**
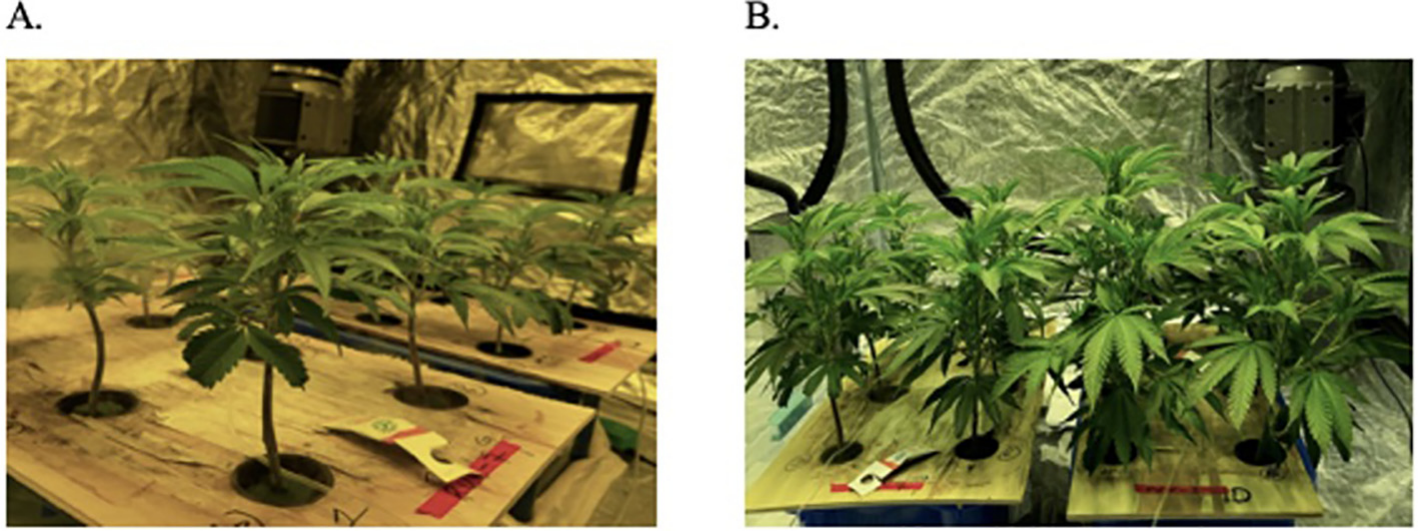
Deep-water culture units showing *C*. *sativa* plants **(A)** 7 days and **(B)** 14 days after propagation.

The medical cannabis accession “The New” (Lyonleaf, Montreal, Qc, Canada) was used as a model plant for this study. Consistent 2-week-old clones (approximately 15-cm tall, with four to five nodes pruned to four leaves) planted in rockwool cubes (Grodan A-OK Starter Plugs, ROCKWOOL Group, Hedehusene, Denmark) were transplanted into a deep-water culture (DWC) unit using a mesh pot (**Figure 1**). The clones in the mesh pot were submerged 3 cm into the nutrient solution as described previously (Bevan et al., 2021). The plants were grown in DWC systems in a controlled environment using a growing tent and 18/6-h light/dark photoperiod for 2 weeks using broad amber light (5,000 K; U Technology Corporation, Calgary, Canada), with an intensity range of 360–400 µmol m^-2^ s^-1^. Most medical cannabis producers limit the length of the vegetative stage (2 to 3 weeks) to control the size of the mature plant and improve the consistency of secondary metabolites in the plant (Saloner and Bernstein, 2020). Each DWC unit consisted of a 15-L hydroponic system basin with the nutrient solution and an aerator (Pawfly, Naludo-NL138, Snapklik, New York, USA) to mix and aerate the solution continually. The nutrient solutions in all DWC units were drained and replaced with 15 L of fresh nutrient solutions weekly. The relative humidity (RH), air temperature, and vapor pressure deficit (VPD) in the growing tent were maintained at 65 ± 5%, 25 ± 1°C, and 1.11 ± 0.25 kPa, respectively. The environmental CO_2_ in the growing tent was measured with an air quality monitor (LC-1038, Langkou, Shandong, China) and ranged between 470 and 520 ppm. The initial and final nutrient solution temperatures were 20°C and 24°C, respectively (HANNA Instruments, Laval, Quebec, Canada).

### 2.2 Experimental design and nutrient solution treatments

A uniformly precise five-level by three-variable central composite rotatable statistical design (CCRD) was employed to model the cannabis growth responses to the different primary macronutrients (NPK). The experimental design consisted of 20 combinations of the independent variables. These were based on previous studies with reported concentrations of 160–240 mg L^−1^ N (Saloner and Bernstein, 2021), 30–90 mg L^−1^ P (Shiponi and Bernstein, 2021), and 60–175 mg L^−1^ (Saloner et al., 2019).

The levels of the independent variables chosen were coded as -1.682, -1, 0, + 1, and +1.682. The complete CCRD experimental matrix is presented in **Table 1**. The first eight combinations of values of the independent variables correspond to a standard 2*^k^* factorial (where *k* is the number of variables) and coded as +1 and -1. The next set of six combinations constitutes 2k points, known as the axial points. They were fixed at a distance of 1.682 (*α* = $2^{k/4}$ ) from the center to ensure rotatability (Mead, 1988). Axial points were utilized to incorporate quadratic terms into the response surface model. The last six rows in the matrix form the center points and were coded 0. There were 15 different treatments with five replicates per treatment. Replication of the experimental run at the center in space and time assured increased consistency and greater uniformity in the precision of response estimation across the experimental domain (Bevan et al., 2021; Addo et al., 2022a; Addo et al., 2022b). **Table 2** shows a list of the primary cations and anions that are present in each of the treatment solutions.

**Table 1:** Experimental factors’ range and levels according to a three-factor central rotatable composite design.

Treatments	Nitrogen (mg L^-1^)	Phosphorus (mg L^-1^)	Potassium (mg L^-1^)
1	160 (-1)	30 (-1)	60 (-1)
2	160 (-1)	30 (-1)	175 (1)
3	160 (-1)	90 (1)	60 (-1)
4	160 (-1)	90 (1)	175 (1)
5	240 (1)	30 (-1)	60 (-1)
6	240 (1)	30 (-1)	175 (1)
7	240 (1)	90 (1)	60 (-1)
8	240 (1)	90 (1)	175 (1)
9	132.7 (-1.682)	60 (0)	117.5 (0)
10	267.3 (-1.682)	60 (0)	117.5 (0)
11	200 (0)	9.6 (-1.682)	117.5 (0)
12	200 (0)	110.5 (-1.682)	117.5 (0)
13	200 (0)	60 (0)	20.8 (-1.682)
14	200 (0)	60 (0)	214.2 (-1.682)
15	200 (0)	60 (0)	117.5 (0)
16	200 (0)	60 (0)	117.5 (0)
17	200 (0)	60 (0)	117.5 (0)
18	200 (0)	60 (0)	117.5 (0)
19	200 (0)	60 (0)	117.5 (0)
20	200 (0)	60 (0)	117.5 (0)

Coded values are in parentheses.

**Table 2:** Composition of major cations and anions in the treatment nutrient solutions.

Treatment	Nitrogen (mg L^-1^)	Phosphorus (mg L^-1^)	Potassium (mg L^-1^)	Sulphur (mg L^-1^)	Magnesium (mg L^-1^)	Calcium (mg L^-1^)	Sodium (mg L^-1^)
1	160	30	60	101.6	77	435.3	
2	160	30	175	101.6	77	317.5	
3	160	90	60	101.6	77	435.3	44.5
4	160	90	175	101.6	77	395.2	
5	240	30	60	101.6	77	664.5	
6	240	30	175	101.6	77	546.7	
7	240	90	60	101.6	77	665.3	44.5
8	240	90	175	101.6	77	664.5	
9	132.7	60	117.5	101.6	77	337.1	
10	267.3	60	117.5	101.6	77	722.2	
11	200	9.6	117.5	101.6	77	464.5	
12	200	110.5	117.5	101.6	77	490	59.7
13	200	60	20.8	101.6	77	551.1	44.5
14	200	60	214.2	101.6	77	430.4	
15	200	60	117.5	101.6	77	529	
16	200	60	117.5	101.6	77	529	
17	200	60	117.5	101.6	77	529	
18	200	60	117.5	101.6	77	529	
19	200	60	117.5	101.6	77	529	
20	200	60	117.5	101.6	77	529	

Treatment combinations were defined by their concentrations of N (132.7, 160, 200, 240, and 267.3 mg L^-1^), P (9.6, 30, 60, 90, and 110.5 mg L^-1^), and K (20.8, 60, 117.5, 175, and 214.2 mg L^-1^). The nutrient solutions were prepared with distilled water using KNO_3_ (Haifa-Group, Matam-Haifa, Israel), Ca(NO_3_)_2_*4H_2_O, KH_2_PO_4_, MgSO_4,_ and NaH_2_PO_4_*H_2_O (Fisher Scientific, Ottawa, Ontario, Canada). Every nutrient treatment group had six plants assigned to it at random. Every treatment was given the same amount of Hoagland solution recipes B (boron, manganese, zinc, copper, and molybdenum) and C (iron and ethylene diamine tetraacetic acid) (Hoagland and Arnon, 1950). The initial pH of the nutrient treatments was adjusted to 5.8 with 1 M H_2_SO_4_ or 1 M NaOH as needed. The pH and electrical conductivity (EC) of the nutrient solution were measured before and after the treatments using hand-held pH and EC meters (HANNA Instruments, Laval, Quebec, Canada; **Supplementary Table S1**). The photosynthetic photon flux density (PPFD) at the top of each plant was determined at the beginning, middle, and end of the experimental runs using a light meter (LI-250A; LI-COR Inc., Lincoln, NE, USA) (Mansoori et al., 2023). Light mapping was performed to ensure that each cannabis plant received consistent PPFD during its growth and between replicated runs.

### 2.3 Plant growth measurements

Plant height (H) (cm) was measured from the top of the rockwool to the top of the apical bud of the plant. Plant width (cm) was measured at the widest point on the plant (width 1) and then perpendicular to this measurement (width 2). The growth index (GI) was calculated using Equation 1 as described previously (Ruter, 1992; Caplan et al., 2017b).


(1) $$Growth\;index=\frac{height\;x\;width\;1\;x\;width\;2}{300}$$

Stem diameter (SD) was measured 3 cm from the top of the rockwool using a carbon fiber composite digital caliper (Dasqua 2220-8113, Chengdu, Sichuan, China). Leaf area (LA) was measured using imaging software (ImageJ 1.48v, Bethesda, MD, USA). Fresh roots, stems, and leaves were measured using Valor 3000 Xtreme Portable Scale (ITM Instruments, Quebec, Canada) to determine the fresh mass (FM) of each treatment. To determine the dry mass (DM), biomass was dried in an Isotemp oven (Fisher Scientific, Quebec, Canada) at 65°C for 24 h. The ratio of leaf area to dry leaf mass was used to compute the specific leaf area (SLA) (Mansoori et al., 2023). Nutrient use efficiency was calculated from the ratio of plant yield (dry mass) to the input of the nutrient, expressed as a percentage (Fageria, 2012). Fresh cannabis leaf samples (50 g) were sent to the University of Guelph’s Agriculture and Food Laboratory (Guelph, Ontario, Canada) for elemental analysis.

The youngest mature fan leaf was used to measure the amounts of chlorophyll a and b using a previously reported methodology (Gorelick et al., 2019). Chlorophyll a and b were calculated using Equations 2 and 3 as described previously (Lichtenthaler and Wellburn, 1983).


(2) $$Chlorophyll_{a}=12.21A_{663}–2.81A_{646}$$

(3) $$Chlorophyll_{b}=\;20.13A_{646}–\;5.03A_{663}$$

### 2.4 Statistical analysis

JMP software (JMP 4.3 SAS Institute, Inc.) was used for data analysis. The relationship between the independent and dependent variables was evaluated using the least square multiple regression methodology (LSMR). Based on the experimental data, the multiple regression equation was used to fit the second-order polynomial model. This model includes all the independent variables and their respective quadratic and interaction terms (Equation 4). By performing Fisher’s F-test at 95% confidence level, the statistical significance of the regression coefficients was examined using analysis of variance (ANOVA). The quality of fit of each model to the responses was estimated using the correlation coefficient (*R* ^2^). JMP software was used to create response representations in three dimensions using surface plots. These plots were used to determine the optimal and the maximum desired rate of all three factors.


(4) $$Y_{j}=b_{0}+\;n\;+\;n^{2}+\;p\;+\;p^{2}+\;k\;+\;k^{2}+\;\left(n\;x\;p\right)\;+\;\left(n\;x\;k\right)\;+\;\left(p\;x\;k\right)\;+\;\left(n\;x\;p\;x\;k\right)$$

Y_j_ = predicted response of the dependent variables

β_0_ = model intercept

n = linear nitrogen component

n^2^ = quadratic nitrogen component

p = linear phosphorus component

p^2^ = quadratic phosphorus component

k = linear potassium component

k^2^ = quadratic potassium component

n × p = nitrogen and phosphorus interaction effect

n × k = nitrogen and potassium interaction effect

p × k = phosphorus and potassium interaction effect

n × p × k = nitrogen, phosphorus, and potassium interaction effect

## 3 Results

This study aimed to evaluate the nutrient interaction effects of the primary macronutrients (NPK) on *C. sativa* plants with a CCRD and to assess the optimal and/or desired primary macronutrient levels during the vegetative stage. The impact of primary macronutrients on the vegetative *C. sativa* plant growth parameters, including H, GI, SD, LA, FM, DM, SLA, number of branches, number of leaves, and chlorophyll content, was measured (**Tables 3**, **4**). **Table 5** shows the impacts of the primary macronutrients on nitrogen use efficiency (NUE), phosphorus use efficiency (PUE), and potassium use efficiency (KUE). The statistical analysis and the desired mineral nutrient requirements for *C. sativa* in the vegetative stage are presented in **Tables 6** and **7**, respectively.

**Table 3:** Effects of primary macronutrient concentrations on the means (*n* = 5) of cannabis leaf mass, height, growth index, root mass, leaf area, and specific leaf area.

Run	Concentration of nutrient (mg L^-1^)			Fresh leaf mass (g)	Dry leaf mass (g)	Height (cm)	Growth index	Fresh root mass (g)	Dry root mass (g)	Leaf area (cm^2^)	Specific leaf area (cm^2^ g^-1^)
	Nitrogen	Phosphorus	Potassium								
1	160	30	60	0.7	0.15	21.5	67.1	10.1	0.43	47.1	317.4
2	160	30	175	0.86	0.18	22	118.2	11.8	0.5	87.7	484.8
3	160	90	60	2.09	0.63	22	122.4	17.6	1.07	99.3	158.5
4	160	90	175	0.80	0.15	22.7	121.4	10.6	0.5	86.9	585.7
5	240	30	60	0.82	0.16	20.4	110.9	13.8	0.58	49.9	308.2
6	240	30	175	1.29	0.33	17.7	63.2	9.79	0.43	63.2	189.7
7	240	90	60	1.9	0.61	18.2	90.3	17.1	1.08	86.2	141.9
8	240	90	175	1.38	0.38	17.5	58.1	11.7	0.51	78.8	209.3
9	132.7	60	117.5	1.38	0.36	21.4	91.6	11.9	0.51	78.1	219.1
10	267.3	60	117.5	1.18	0.31	18.6	67.6	9.48	0.43	73.8	237.3
11	200	9.6	117.5	0.59	0.18	12.2	20.6	6.62	0.29	33.9	192.8
12	200	110.5	117.5	1.89	0.58	24.2	157.1	22.7	1.36	87.1	151.8
13	200	60	20.8	1.36	0.47	20.7	97.6	15.4	1.01	48.1	100.6
14	200	60	214.2	0.87	0.25	15.4	47	9.01	0.36	54.1	219.8
15	200	60	117.5	1.05	0.28	20.5	60.7	10.1	0.47	57.1	205.1
16	200	60	117.5	1.27	0.34	23	68.1	10.9	0.52	59.4	174.8
17	200	60	117.5	1	0.26	20.2	63.5	10.1	0.47	64.4	247.3
18	200	60	117.5	0.87	0.24	21.2	62.8	9.24	0.42	66.0	278.5
19	200	60	117.5	1.05	0.3	20.2	59.8	10.4	0.49	55.3	183.9
20	200	60	117.5	0.82	0.22	17.7	49.2	9.03	0.38	65.7	297.7

**Table 4:** Effects of primary macronutrient concentrations on the means (*n* = 5) of cannabis stem mass, stem diameter, number of branches, number of leaves, and chlorophyll a and b contents.

Run	Concentration of nutrient (mg L^-1^)			Fresh stem mass (g/cm)	Dry stem mass (g/cm)	Stem diameter (cm)	Number of branches	Number of leaves	Chlorophyll a content (mg g^-1^)	Chlorophyll b content (mg g^-1^)
	Nitrogen	Phosphorus	Potassium							
1	160	30	60	0.17	0.02	0.22	7	24	0.08	0.04
2	160	30	175	0.32	0.04	0.20	8	28	0.14	0.06
3	160	90	60	0.40	0.05	0.42	9	39	0.17	0.07
4	160	90	175	0.26	0.03	0.24	7	27	0.11	0.05
5	240	30	60	0.19	0.03	0.22	7	21	0.08	0.04
6	240	30	175	0.19	0.02	0.26	8	24	0.08	0.03
7	240	90	60	0.37	0.05	0.38	9	33	0.15	0.07
8	240	90	175	0.20	0.02	0.21	7	19	0.08	0.04
9	132.7	60	117.5	0.22	0.02	0.30	8	23	0.09	0.04
10	267.3	60	117.5	0.2	0.02	0.25	8	23	0.09	0.04
11	200	9.6	117.5	0.15	0.02	0.09	6	21	0.07	0.03
12	200	110.5	117.5	0.41	0.05	0.49	12	53	0.16	0.07
13	200	60	20.8	0.3	0.04	0.34	10	43	0.12	0.06
14	200	60	214.2	0.19	0.03	0.16	9	33	0.1	0.04
15	200	60	117.5	0.22	0.03	0.28	9	35	0.09	0.04
16	200	60	117.5	0.23	0.03	0.28	9	30	0.13	0.08
17	200	60	117.5	0.22	0.03	0.23	9	40	0.12	0.07
18	200	60	117.5	0.22	0.03	0.28	9	29	0.12	0.07
19	200	60	117.5	0.22	0.03	0.38	10	38	0.12	0.07
20	200	60	117.5	0.2	0.02	0.23	8	27	0.11	0.08

**Table 5:** Effects of primary macronutrients on nitrogen, phosphorus, and potassium use efficiency.

Run	Concentration of nutrient (mg L^-1^)			Nitrogen use efficiency (%)	Phosphorus use efficiency (%)	Potassium use efficiency (%)
	Nitrogen	Phosphorus	Potassium			
1	160	30	60	0.37	1.98	0.99
2	160	30	175	0.45	2.42	0.42
3	160	90	60	1.09	1.94	2.91
4	160	90	175	0.43	0.76	0.39
5	240	30	60	0.32	2.57	1.29
6	240	30	175	0.33	2.62	0.45
7	240	90	60	0.72	1.93	2.90
8	240	90	175	0.38	1.01	0.52
9	132.7	60	117.5	0.68	1.49	0.76
10	267.3	60	117.5	0.29	8.02	0.66
11	200	9.6	117.5	0.24	0.44	0.41
12	200	110.5	117.5	0.99	3.31	1.69
13	200	60	20.8	0.76	2.54	7.34
14	200	60	214.2	0.31	1.04	0.29
15	200	60	117.5	0.39	1.29	0.66
16	200	60	117.5	0.44	1.47	0.75
17	200	60	117.5	0.38	1.26	0.64
18	200	60	117.5	0.34	1.14	0.58
19	200	60	117.5	0.41	1.36	0.69
20	200	60	117.5	0.31	1.04	0.53

**Table 6:** Statistical analysis (Prob >F) for cannabis growth attributes.

Growth (dependent) parameters	N	P	K	N^2^	P^2^	K^2^	N*P	N*K	K*P	N*P*K
	Prob >F for independent parameters									
Height	0.094	0.092	0.317	0.829	0.529	0.511	0.659	0.575	0.769	0.459
Growth index	0.142	0.017	0.242	0.186	0.084	0.329	0.261	0.095	0.615	0.08
Fresh root mass	0.828	0.0009	0.0119	0.643	0.017	0.198	0.851	0.526	0.142	0.0109
Dry root mass	0.925	<.0001	0.0002	0.953	0.002	0.035	0.838	0.491	0.007	0.0002
Fresh leaf mass	0.343	<.0001	0.0082	0.053	0.086	0.383	0.723	0.045	0.0004	0.0003
Dry leaf mass	0.149	<.0001	0.001	0.347	0.067	0.135	0.777	0.0296	0.0001	<.0001
Fresh stem mass	0.04	<.0001	0.0072	0.932	0.0073	0.116	0.738	0.0424	0.0002	0.0001
Dry stem mass	0.144	<.0001	0.0022	0.748	0.0006	0.0061	0.859	0.0076	0.0002	<.0001
Stem diameter	0.693	0.0009	0.015	0.859	0.858	0.495	0.432	0.743	0.053	0.02
Number of branches	0.877	0.022	0.372	0.076	0.322	0.830	0.904	1.000	0.204	0.23
Number of leaves	0.374	0.011	0.160	0.019	0.902	0.747	0.674	0.833	0.096	0.07
Chlorophyll a content	0.119	0.001	0.107	0.102	0.930	0.905	0.784	0.146	0.0035	0.008
Chlorophyll b content	0.352	0.0219	0.253	0.0147	0.139	0.114	0.851	0.329	0.0477	0.045
Leaf area	0.167	0.0002	0.216	0.0088	0.357	0.678	0.984	0.408	0.0165	0.004
Specific leaf area	0.0843	0.450	0.058	0.371	0.891	0.975	0.745	0.0358	0.125	0.038
Nitrogen use efficiency	0.0019	<.0001	0.0002	0.230	0.0052	0.0496	0.315	0.314	0.0007	<.0001
Phosphorus use efficiency	0.0464	0.872	0.453	0.0215	0.988	0.954	0.894	0.973	0.536	<.0001
Potassium use efficiency	0.935	0.100	0.0002	0.591	0.988	0.0015	0.930	0.961	0.180	<.0001

**Table 7:** Desired mineral nutrient concentrations for cannabis plant growth parameters during the vegetative stage.

Growth parameters	N (mg L^-1^)	P (mg L^-1^)	K (mg L^-1^)	Desirability	Correlation
Fresh leaf mass	160	90	60	0.97	0.96
Dry leaf mass	160	90	60	0.99	0.97
Fresh stem mass	160	90	60	0.93	0.96
Dry stem mass	160	90	60	0.99	0.97
Stem diameter	160	90	60	0.90	0.89
Leaf area	160	90	60	0.83	0.92
Nitrogen use efficiency	160	90	60	0.97	0.97
Height	160	90	123.7	0.88	0.70
Growth index	160	90	175	0.78	0.83
Number of leaves	191.1	90	60	0.77	0.84
Number of branches	199.4	90	60	0.72	0.77
Specific leaf area	200	90	60	0.76	0.82
Chlorophyll a content	203.6	90	60	0.95	0.91
Chlorophyll b content	200	90	60	0.99	0.86
Fresh root mass	240	90	60	0.73	0.90
Dry root mass	240	90	60	0.81	0.96

An increase in [N] (132.7–267.3 mg L^-1^ and 160–240 mg L^-1^) at different concentrations of P and K resulted in a decrease in H and number of leaves. An increase in [P] (30–90 mg L^-1^) at different concentrations of N and K increased all the plant growth parameters except H and GI. An increase in [K] (20.8–214.2 mg L^-1^ and 60–175 mg L^-1^) at different [N] and [P] resulted in a decrease in SD. However, this trend was not observed when [N] and [P] concentrations were set at 240 and 30 mg L^-1^, respectively. Variable effects on plant growth parameters were observed with increasing [N] and [K] due to the interactive effects of different levels of the macronutrients. Plant growth parameters responded significantly to the linear and quadratic components of N, P, and K.

The concentration of the macronutrients in the cannabis leaves is presented in **Table 8**. Optimum concentrations were observed in N, P, K, calcium (Ca), and sulfur (S). Three-dimensional response surface plots indicate that an increase in [P] and [K] decreased the concentration of Mg while it was unaffected by the increase in [N].

**Table 8:** Macronutrient concentration of cannabis leaves.

Run	Nitrogen (mg g^-1^)	Phosphorus (mg g^-1^)	Potassium (mg g^-1^)	Calcium (mg g^-1^)	Magnesium (mg g^-1^)	Sulfur (mg g^-1^)
1	0.507	0.0715	0.271	0.474	0.0538	3.7
2	0.502	0.0642	0.284	0.475	0.0611	3.6
3	0.516	0.06	0.162	0.522	0.0559	3.8
4	0.513	0.0668	0.212	0.486	0.0496	3.4
5	0.528	0.0619	0.262	0.535	0.0399	3.8
6	0.538	0.0705	0.279	0.508	0.0453	3.8
7	0.509	0.0629	0.175	0.568	0.04	3.6
8	0.544	0.0717	0.254	0.501	0.0407	3.8
9	0.523	0.0712	0.26	0.502	0.0622	3.9
10	0.528	0.0707	0.26	0.524	0.0373	3.6
11	0.45	0.0676	0.257	0.427	0.048	3.6
12	0.521	0.0654	0.261	0.53	0.0462	3.7
13	0.486	0.0598	0.116	0.508	0.0463	3.5
14	0.485	0.0701	0.271	0.466	0.0517	3.8
15	0.538	0.0714	0.273	0.549	0.0468	3.9
16	0.545	0.0723	0.281	0.553	0.047	3.8
17	0.543	0.0729	0.277	0.552	0.0469	3.8
18	0.558	0.0743	0.273	0.555	0.0474	3.8
19	0.552	0.0739	0.284	0.56	0.0475	3.9
20	0.535	0.0712	0.271	0.542	0.0464	3.8

### 3.1 Effects of N on plant growth parameters

**Tables 3** and **4** indicate that increasing [N] from 160 to 240 mg L^-1^ at [P] and [K] of 30 and 60 mg L^-1^, respectively, decreased the H (5.1%) and number of leaves (12.5%). At the same [P] and [K], an increase in [N] resulted in increased GI (65.3%), fresh root mass (36.6%), dry root mass (34.9%), fresh leaf mass (17.1%), dry leaf mass (6.7%), LA (5.9%), fresh stem mass (11.8%), and dry stem mass (50.0%), although there was no change in the SD, number of branches, chlorophyll a content, and chlorophyll b content.

An increase in [N] from 160 to 240 mg L^-1^ at [P] and [K] of 30 and 175 mg L^-1^, respectively, resulted in a decrease in H, GI, fresh root mass, dry root mass, fresh stem mass, dry stem mass, chlorophyll a content, chlorophyll b content, number of leaves, and LA by 19.5%, 46.5%, 17.0%, 14.0%, 40.6%, 50.0%, 42.9%, 50.0%, 14.3%, and 27.9%. There was no change in the number of branches, although fresh leaf mass, dry leaf mass, and SD increased by 50.0%, 83.3%, and 30.0%, respectively.

An increase in [N] from 160 to 240 mg L^-1^ at P and K concentrations of 90 and 175 mg L^-1^, respectively, decreased H (22.9%), GI (52.1%), fresh stem mass (23.1%), dry stem mass (33.3%), SD (12.5%), number of leaves (29.6%), chlorophyll a content (27.3%), chlorophyll b content (20.0%), and LA (9.3%). There was no change in the number of branches, although fresh root mass, dry root mass, fresh leaf mass, and dry leaf mass increased by 10.4%, 2.0%, 72.5%, and 153.3%, respectively.

An increase in [N] from 132.7 to 267.3 mg L^-1^ at [P] and [K] of 60 and 117.5 mg L^-1^, respectively, decreased H (13.1%), GI (26.2%), fresh root mass (20.3%), dry root mass (15.7%), fresh stem mass (9.1%), SD (16.7%), fresh leaf mass (14.5%), dry leaf mass (13.9%), and LA (5.5%). There was no change in the number of branches, number of leaves, dry stem mass, and chlorophyll a and b contents.

Statistical analyses and modeling (**Table 6**) showed that N had a significant effect (*p* < 0.05) on fresh stem mass and NUE. The quadratic component of N responded significantly (*p* < 0.05) to LA, PUE, chlorophyll b content, and the number of leaves.

### 3.2 Effects of P on plant growth parameters

**Tables 3** and **4** showed that an increase in [P] from 30 to 90 mg L^-1^ at [N] and [K] of 160 and 60 mg L^-1^, respectively, increased H (2.3%), GI (82.4%), fresh root mass (74.3%), dry root mass (148.8%), fresh leaf mass (198.6%), dry leaf mass (320%), fresh stem mass (135.3%), dry stem mass (150.0%), SD (90.9%), number of branches (28.6%), number of leaves (62.5%), chlorophyll a content (112.5%), chlorophyll b content (75.0%), and LA (110.8%).

An increase in [P] 30 to 90 mg L^-1^ at [N] and [K] of 240 and 60 mg L^-1^, respectively, increased fresh root mass (23.9%), dry root mass (86.2%), fresh leaf mass (131.7%), dry leaf mass (281.3%), fresh stem mass (94.7%), dry stem mass (66.7%), SD (72.7%), number of branches (28.6%), number of leaves (57.1%), chlorophyll a content (87.5%), chlorophyll b content (75.0%), LA (72.7%), and SLA (75.0%). H and GI decreased by 10.8% and 18.6%, respectively.

Statistical analyses and modeling (**Table 6**) showed that P had a significant effect (*p* < 0.05) on all the growth parameters (GI, SD, LA, fresh root mass, dry root mass, fresh leaf mass, dry leaf mass, fresh stem mass, dry stem mass, number of branches, number of leaves, and NUE) except H, SLA, PUE, and KUE. The quadratic component of P responded significantly (*p* < 0.05) to fresh root mass, dry root mass, fresh stem mass, and dry stem mass.

### 3.3 Effects of K on plant growth parameters

**Tables 3** and **4** indicate that an increase in [K] from 20.8 to 214.2 mg L^-1^ at [N] and [P] of 200 and 60 mg L^-1^, respectively, decreased H (25.6%), GI (51.8%), fresh root mass (41.5%), dry root mass (64.4%), fresh leaf mass (36.0%), dry leaf mass (46.8%), fresh stem mass (36.7%), dry stem mass (25.0%), SD (52.9%), number of branches (10.0%), number of leaves (23.3%), chlorophyll a content (16.7%), and chlorophyll b content (33.3%). LA increased by 12.5%.

An increase in [K] from 60 to 175 mg L^-1^ at [N] and [P] of 240 and 90 mg L^-1^, respectively, decreased P (3.8%), GI (35.7%), fresh root mass (31.6%), dry root mass (52.8%), fresh leaf mass (27.4%), dry leaf mass (37.7%), fresh stem mass (45.9%), dry stem mass (60.0%), SD (44.7%), number of branches (22.2%), number of leaves (42.4%), chlorophyll a content (46.7%), chlorophyll b content (42.9%), and LA (8.6%).

An increase in [K] from 60 to 175 mg L^-1^ at [N] and [P] of 240 and 30 mg L^-1^, respectively, increased fresh leaf mass (57.3%), dry leaf mass (106.3%), SD (18.2%), LA (26.7%), number of branches (14.3%), and number of leaves (14.3%). Fresh stem mass and chlorophyll a content remained unchanged, although H, GI, fresh root mass, dry root mass, and chlorophyll b content decreased by 15.3%, 43.0%, 29.1%, 25.9%, and 25.0%, respectively.

Statistical analyses and modeling (**Table 6**) showed that K had a significant effect (*p* < 0.05) on fresh root mass, dry root mass, fresh leaf mass, dry leaf mass, fresh stem mass, dry stem mass, SD, NUE, and KUE. The quadratic component of K responded significantly (*p* < 0.05) to dry root mass, stem mass, and KUE.

### 3.4 Interaction effect of N, P, and K on growth parameters

**Figure 2:**
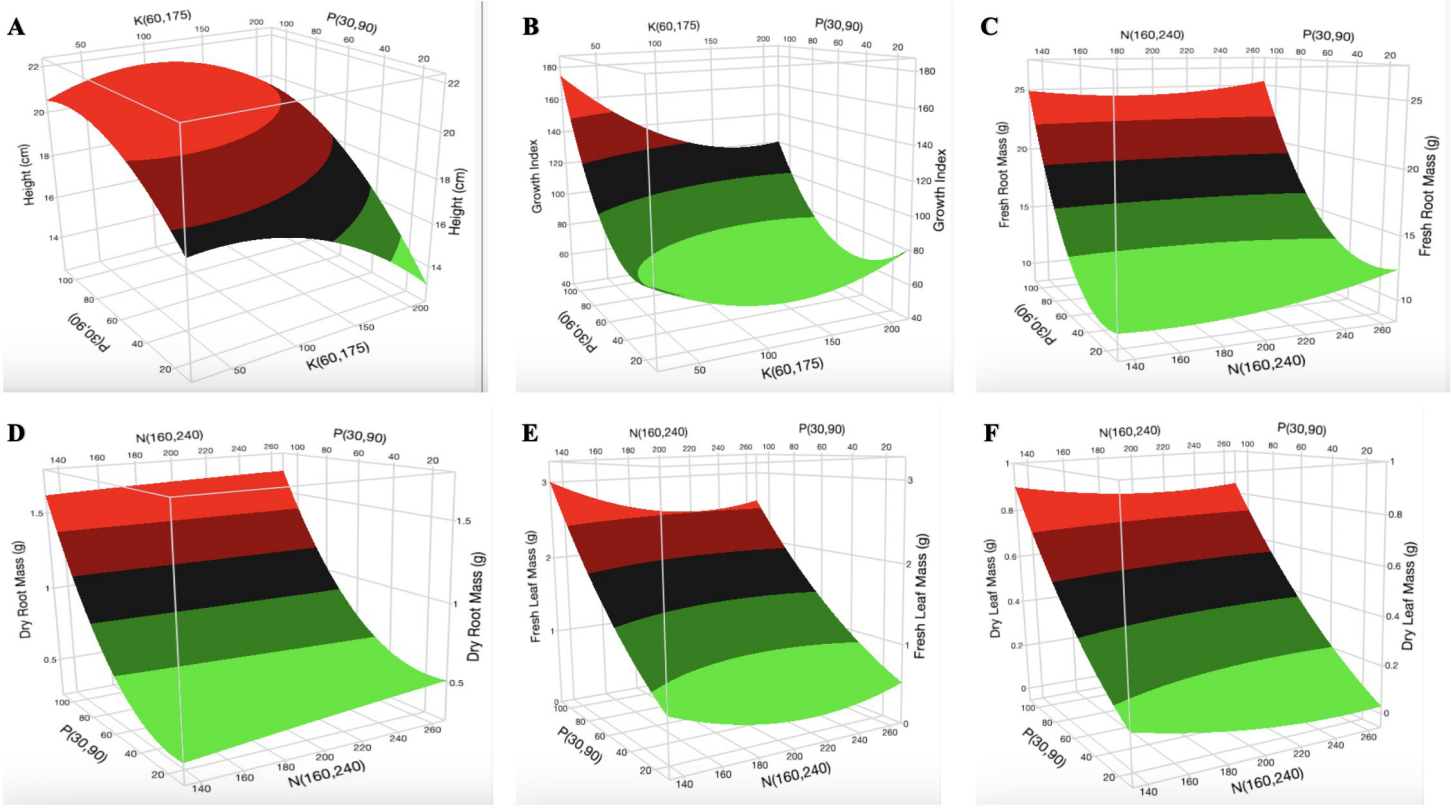
Three-dimensional response surface responses for the effect of nitrogen and phosphorus on **(A)** plant height, **(B)** growth index, **(C)** fresh root mass, **(D)** dry root mass, **(E)** fresh leaf mass, and **(F)** dry leaf mass at various treatments in the deep-water culture system.

**Figure 3:**
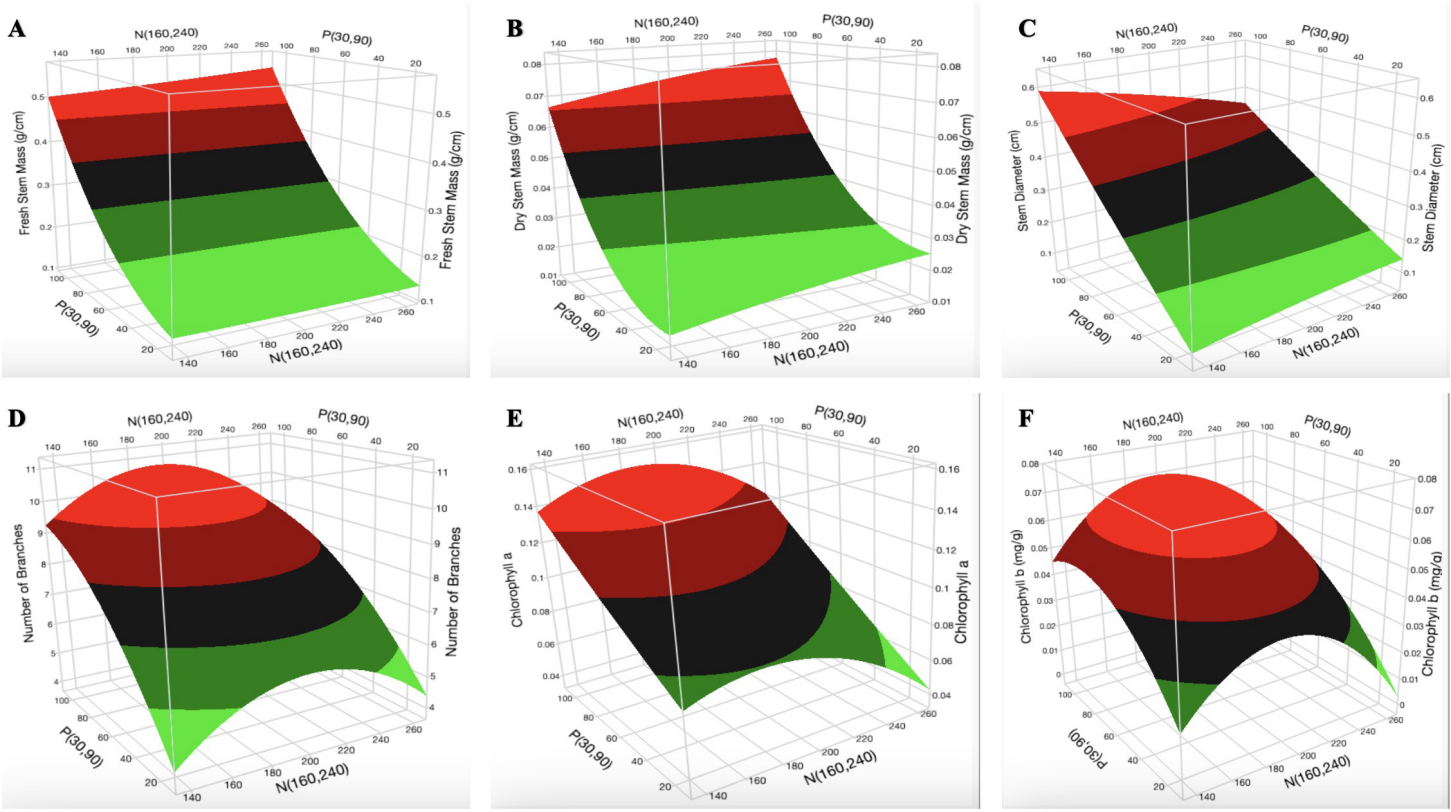
Three-dimensional response surface responses for the effect of nitrogen and phosphorus on **(A)** fresh stem mass, **(B)** dry stem mass, **(C)** stem diameter, **(D)** number of branches, **(E)** chlorophyll a content, and **(F)** chlorophyll b content at various treatments in the deep-water culture system.

Constructed 3D surface plots of K vs. P at N = 160 mg L^-1^ for H (**Figure 2A**) show that the optimal [K] is in the range of 70–130 mg L^-1^. The surface plot of K vs. N at P = 90 mg L^-1^ for H (**Supplementary Figure S1B**) shows that the optimal [N] is in the range of 140–200 mg L^-1^. Optimization was not observed for P. The surface plot of K vs. P at N = 160 mg L^-1^ (**Figure 2B**) and N vs. P at K = 175 mg L^-1^ (**Supplementary Figure 2A**) indicates a decrease in GI with increasing [K] and [N], respectively. P had a non-linear effect (biphasic response) on the GI. As shown in **Figure 2B** and **Supplementary Figures 2A** and **B**, optimization was not observed for the GI. An increase in [N] did not have a major impact on the fresh root mass and dry root mass (**Figures 2C, D**). An increase in [P] and [K] resulted in an increase and a decrease in fresh root mass and dry root mass, respectively (**Supplementary Figures S3B**, **S4A**). An increase in [P] increased the fresh leaf mass and dry leaf mass (**Figures 2E, F**). An increase in [N] resulted in a slight decrease in fresh leaf mass but was not very evident in the dry leaf mass. The surface plot of N vs. P at K = 60 mg L^-1^ indicates an increase in fresh stem mass and dry stem mass with increasing [N] and [P] (**Figures 3A, B**). The surface plot of N vs. P at K = 60 mg L^-1^ indicates that an increase in [P] increased the SD while an increase in [N] resulted in a reduction in SD (**Figure 3C**). The surface plot of N vs. K at P = 90 mg L^-1^ indicated that K did not have a major impact on the SD (**Supplementary Figure S9A**). A plot of K vs. P at N = 160 mg L^-1^ shows that an increase in [K] results in a decrease in SD (**Supplementary Figure S9B**). The surface plots of N vs. P at K = 60 mg L^-1^ show that the optimization range for the number of branches, chlorophyll a content, and chlorophyll b content are in the range of 180–220 mg L^-1^ N (**Figures 3D–F**). The optimization of [K] from the surface plot of N vs. K at P = 90 mg L^-1^ for the number of branches (**Supplementary Figure S10A**) and chlorophyll b content (**Supplementary Figure S13A**) is in the range of 100–150 and 70–200 mg L^-1^ K, respectively.

**Figure 4:**
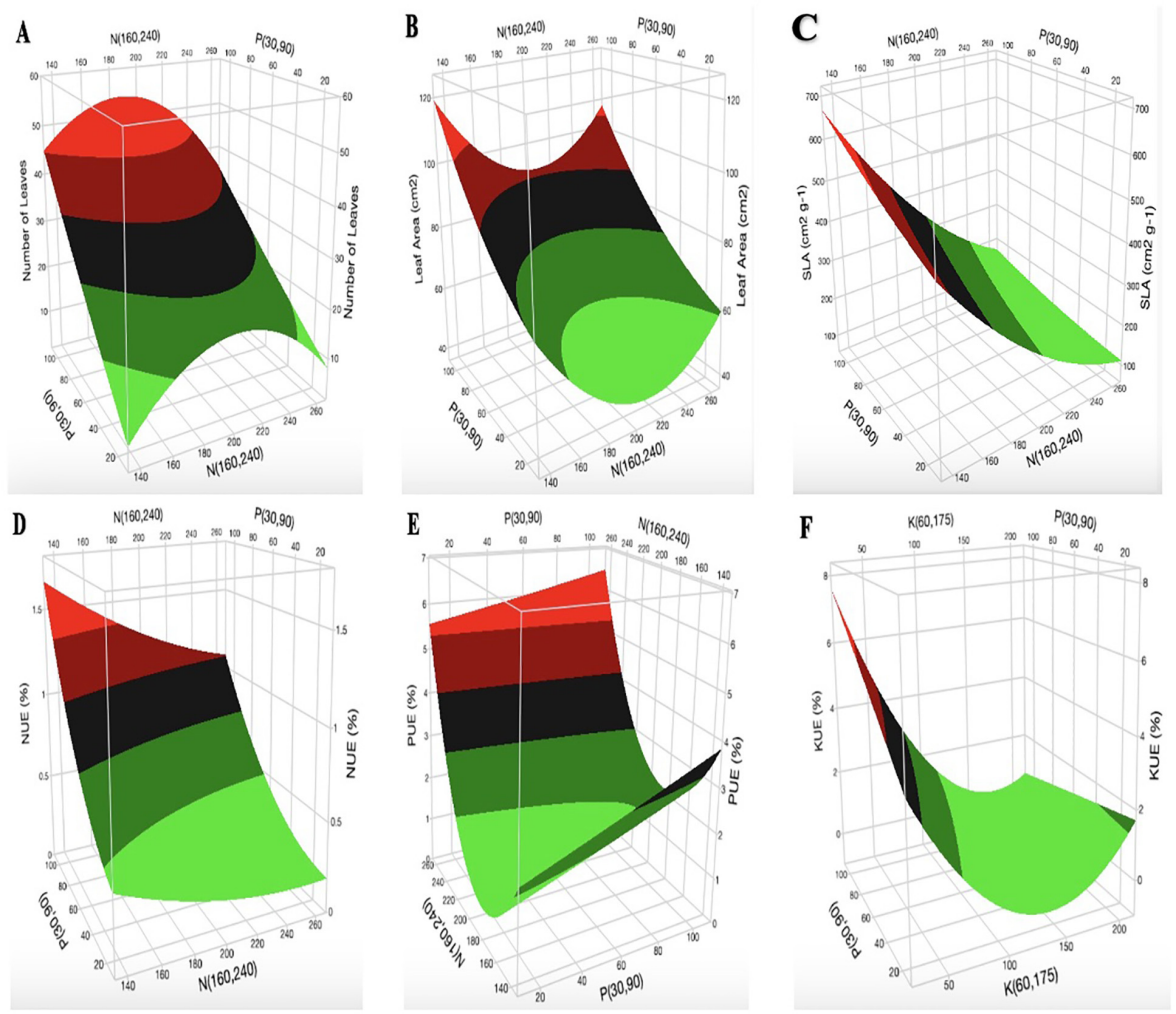
Three-dimensional response surface responses for the effect of nitrogen, phosphorus, and potassium on **(A)** number of leaves, **(B)** leaf area, **(C)** specific leaf area, **(D)** nitrogen use efficiency, **(E)** phosphorus use efficiency, and **(F)** potassium use efficiency at various treatments in the deep-water culture system.

The surface plots of N vs. P at K = 60 mg L^-1^ indicate that the optimal range for the number of cannabis leaves is 180–220 mg L^-1^ N (**Figure 4A**). The optimization of [K] from the surface plot of N vs. K at P = 90 mg L^-1^ for the number of leaves (**Supplementary Figure S11**) falls within the range of 100–170 mg L^-1^. An increase in [N] initially resulted in a decrease in LA (**Figure 4B**; **Supplementary Figure S14A**). However, when [N] exceeds 200 mg L^-1^, LA begins to increase. The surface plot of K vs. P at N = 160 mg L^-1^ shows a marginal decrease in LA with increasing [K]. The increase in [P] did not show any major impact on LA (**Supplementary Figure S14B**). The surface plot of N vs. P at K = 175 mg L^-1^ shows a decrease in SLA with an increase in [N] (**Figure 4C**). The same observation was seen in the surface plot of N vs. K at P = 90 mg L^-1^ for SLA (**Supplementary Figure S15A**). However, the surface plot of K vs. P at N = 160 mg L^-1^ shows an increase in SLA with an increase in [P] (**Supplementary Figure S15B**). The surface plots of N vs. P at K = 60 mg L^-1^ show that an increase in [K] and [P] results in a decrease and an increase in NUE, respectively (**Figure 4D**). The results from N vs. K at P = 90 mg L^-1^ show that an increase in [N] increases NUE (**Supplementary Figure S16A**). An increase in [P] from 20 mg L^-1^ to 100 mg L^-1^ increased PUE by 1% (**Figure 4E**). An increase in [K] decreased PUE, as shown in **Supplementary Figure S17A**. As presented in **Figure 4F**, an increase in [K] decreased KUE. Optimal KUE falls within the range of 180–200 mg L^-1^ [N] (**Supplementary Figure S18A**).

Statistical analyses and modeling (**Table 6**) showed that the N × K interaction had a significant effect (*p* < 0.05) on fresh leaf mass, dry leaf mass, fresh stem mass, dry stem mass, and SLA. The K × P interaction had a significant effect (*p* < 0.05) on dry root mass, fresh leaf mass, dry leaf mass, fresh stem mass, dry stem mass, chlorophyll a content, LA, and NUE. The N × P × K interaction had significant effects (*p* < 0.05) on fresh root mass, dry root mass, fresh leaf mass, dry leaf mass, fresh stem mass, dry stem mass, SD, chlorophyll a and b contents, LA, SLA, NUE, PUE, and KUE. Although there was no significant interaction effect for N × P, N × K, and K × P on the plant physiological traits such as fresh root mass, SD, and number of leaves, a significant interaction effect was observed for N × P × K. All plant growth parameters except H, GI, number of branches, NUE, PUE, and KUE were significantly influenced by the interaction effect of N × P × K.

### 3.5 Mineral composition in cannabis leaf

**Figure 5:**
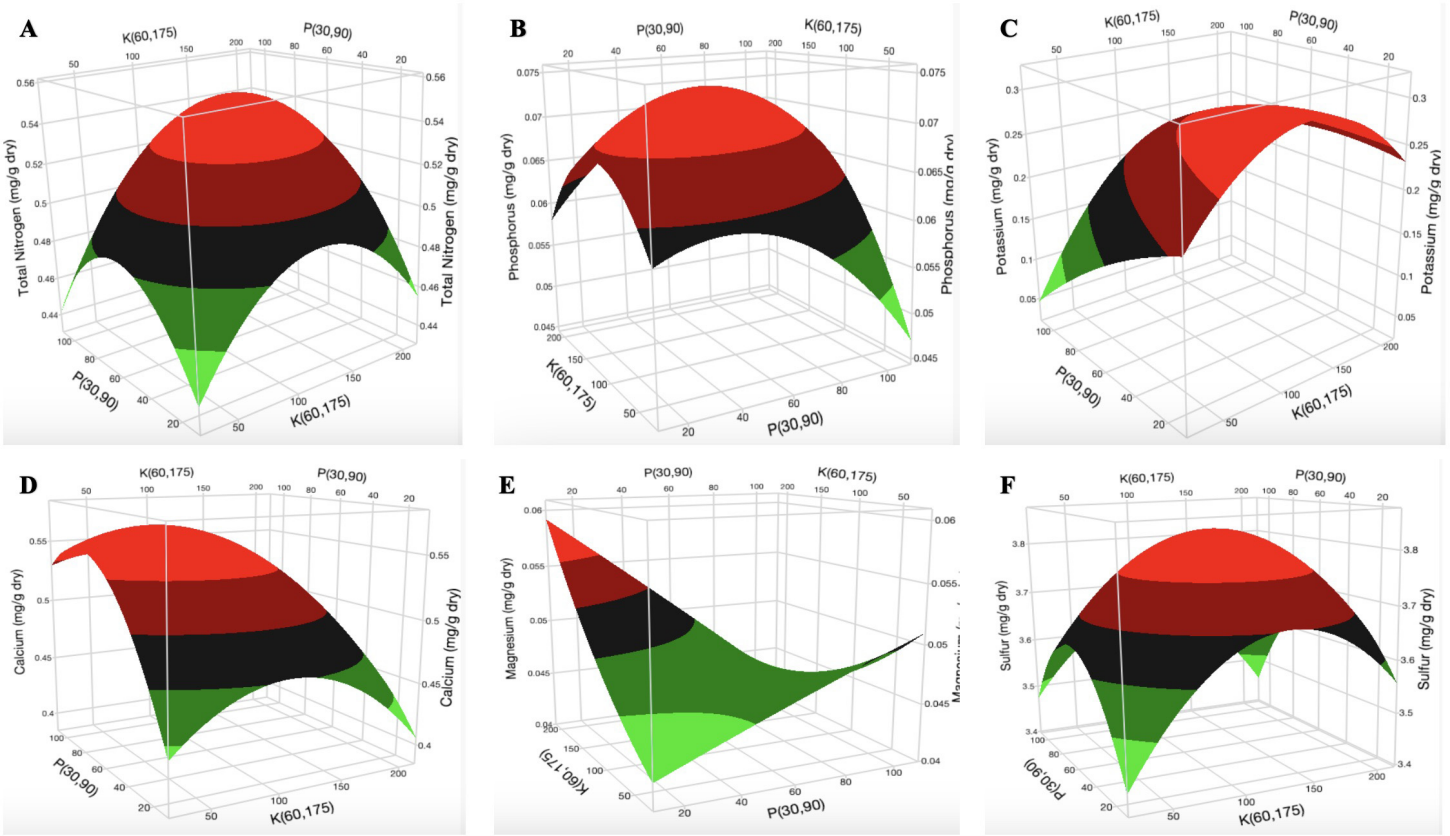
Three-dimensional response surface responses of **(A)** total nitrogen, **(B)** phosphorus, **(C)** potassium, **(D)** calcium, **(E)** magnesium, and **(F)** sulfur content of dry cannabis leaf samples (in mg g^-1^).

The surface plot of K vs P at N = 200 mg L^-1^ shows that the optimal concentration of total nitrogen (TN) content in the cannabis leaf was 0.54 mg g^-1^ at 240–260 mg L^-1^ N, 40–80 mg L^-1^ P, and 160–200 mg L^-1^ K (**Figure 5A**; **Supplementary Figures S19A, B**). The optimal [P] was 0.073 mg g^-1^ at 230–260 mg L^-1^ N, 70–90 mg L^-1^ P, and 160–190 mg L^-1^ K (**Figure 5B**; **Supplementary Figures S20A, B**). The optimal [K] in the cannabis leaf was 0.27 mg g^-1^ at 220–260 mg L^-1^ N, 40–60 mg L^-1^ P, and 150–200 mg L^-1^ K (**Figure 5C**; **Supplementary Figures S21A, B**). The surface plot of K vs P at N = 200 mg L^-1^ indicates that the optimal [Ca] in the cannabis leaf was 0.56 mg g^-1^ at 240–260 mg L^-1^ N, 80 mg L^-1^ P, and 100 mg L^-1^ K (**Figure 5D**; **Supplementary Figures S22A, B**). The surface plot of P vs K at N = 200 mg L^-1^ shows that an increase in [P] reduced the [Mg] in the cannabis leaf (**Figure 5E**). An increase in [N] did not affect [Mg] in the cannabis leaf (**Supplementary Figure S23A**). However, increased [K] results in a decrease in [Mg] from 0.051 mg g^-1^ to 0.044 mg g^-1^ in the cannabis leaf. The [Mg] was the same (0.044 mg g^-1^) between 120 and 200 mg L^-1^ K (**Supplementary Figure S23B**). The optimum [S] in the cannabis leaf was 0.38 mg L^-1^ at 220–260 mg L^-1^ N, 80 mg L^-1^ P, and 150–200 mg L^-1^ K (**Figure 5F**; **Supplementary Figures S24A, B**).

## 4 Discussion

The goal of this study was to evaluate the mineral requirements of N, P, and K in the nutrient solution for the vegetative stage of *C. sativa* in a hydroponic system using RSM. Supplying cannabis plants with the desired amount of mineral nutrients at the vegetative stage can help reduce environmental impact, reduce costs to growers, and promote sustainability without compromising plant growth.

The growth attributes of cannabis plants that enhance their ability to withstand environmental stressors, optimize growth, and build a strong foundation for the flowering stage are H, GI, SD, LA, root development, leaf chlorophyll content, above-dry mass, aboveground plant tissue water content, and specific leaf mass (Moher et al., 2022). For the vegetative parameters of the cannabis plants, there was a significant positive correlation (*r*) between the actual and projected values, ranging from 0.70 to 0.97 (**Table 7**). A perfect positive linear correlation is implied when *r* = 1, and the stronger the positive linear relationship is, the closer *r* is to 1 (Moore and McCabe, 2004).

Responses to mineral nutrition varied between the vegetative growth parameters of the *C. sativa* plant. Findings from this study show that N, P, and K interactions played a significant role in the mineral requirements of the plant. The interactive effect of N × K had a significant impact (*p* < 0.05) on fresh leaf mass, dry leaf mass, fresh stem mass, dry stem mass, and SLA. The K × P interaction had a significant effect (*p* < 0.05) on dry root mass, fresh leaf mass, dry leaf mass, fresh stem mass, dry stem mass, chlorophyll a content, LA, and NUE. N × P × K interaction had a significant effect (*p* < 0.05) on biomass yield, SD, LA, SLA, chlorophyll a and b content, NUE, PUE, and KUE. This observation agrees with previous studies, which show that N interaction with P and/or K helps to improve root development, production of dry matter, and other plant functions that regulate crop yield and quality (Usherwood and Segars, 2001; Milla et al., 2005). The N × P interaction did not have a significant effect (*p* > 0.05) on the growth parameters of the *C. sativa* plant. This observation is in contrast with a previous study, which indicates that N × P interactions can promote nutrient uptake and optimize plant growth (Jiang et al., 2019).

Optimal mineral nutrient concentration was observed in some vegetative parameters of the plants, such as H, number of branches, number of leaves, and chlorophyll a and b contents. The optimal K concentration for H was predicted to be in the range of 100–130 mg L^-1^ in the vegetative stage. This observation aligns with responses observed in other plant species where H was increased due to K application (Besford and Maw, 1975; Asmaa and Hafez, 2010; Zelelew et al., 2016; Kwizera et al., 2019).

In this study, we observed a decrease in H in [K] outside the 100–130 mg L^-1^ K range. The optimal [K] for the number of branches and number of leaves was predicted to be approximately 100–150 and 100–170 mg L^-1^, respectively. This finding aligns with previous studies on *C. sativa*, which reported 60–175 mg L^-1^ as the optimal [K] (Saloner and Bernstein, 2022).

The optimal [N] in the hydroponic solution for the number of branches, number of leaves, and chlorophyll a and b contents was predicted at approximately 199, 191, 204, and 200 mg L^-1^, respectively. This observation aligns with previous studies on other plant species, which showed that the total number of leaves and branches that emerge on a plant and the chlorophyll contents are affected by N fertilization (Vos and van der Putten, 1998; Amanullah et al., 2007; Lee et al., 2011; Ibrahim et al., 2022). We observed a decrease in the number of branches, number of leaves, and chlorophyll a and b contents outside the range of 180–220 mg L^-1^ N.

The optimum range of N for photosynthetic pigments, chlorophyll a and b, falls within the range of 180–220 mg L^-1^. According to one study, the concentration of photosynthetic pigments (chlorophyll a and b content) in *C. sativa* remained constant from 160–240 mg L^-1^ N but increased significantly with an increase in N supply at 30–160 mg L^-1^ N and from 240–320 mg L^-1^ N (Saloner and Bernstein, 2020).

Increased [N] did not have a significant impact on the root mass of the cannabis plant. Previous studies on different plant species show that an increase in [N] inhibited root elongation, thereby reducing root mass (Kwizera et al., 2019). Further studies show that an increase in [N] increased the root mass (Chen et al., 2020). These different observations indicate that [N] might have different impacts on root development in different plant species. Roots provide plants with structural support, nutrition, water, and hormones, all of which directly affect their economic production (Merrill et al., 2002; Fageria and Moreira, 2011). Although roots make up only 10% to 20% of the total plant mass, strong root development is essential for healthy plant growth (Fageria and Moreira, 2011). An increase in [N] resulted in a reduction in fresh leaf mass, but it relatively had no impact on the dry leaf mass of the cannabis plants. This observation might be due to excessive N levels, which might have inhibited leaf expansion due to increased metabolic activity. This phenomenon might not necessarily affect the dry leaf mass of the plant as observed in this study.

The decrease in LA with increasing [N] was followed by an increase in LA with increasing [N] after 200 mg L^-1^ (**Figure 4B**). The initial reduction in LA with increasing N application might be due to N stress or excessive [N]. The sudden increase in LA after 200 mg L^-1^ N might be a coping mechanism for the cannabis plant to adjust and accommodate the excessive [N]. These observations agree satisfactorily with past studies, which indicated that plants adapt their resource allocation and survival strategy, which is reflected in their root and leaves, in response to changes in N nutritional status (Riva et al., 2016). Additional research reveals that whereas root diameter responded more conservatively to N application, an increase in [N] increased leaf length, leaf width, leaf thickness, leaf biomass, and a decrease in SLA (Liu et al., 2023).

An increase in [N] (**Figure 3C**) could potentially lead to a reduction in SD due to decreased lignin content caused by excessive [N]. This observation was reported in two cultivars of winter wheat, where an increase in [N] reduced the SD of the plants (Crook and Ennos, 1995). The increase in stem mass with increasing [N], as observed in this study, is consistent with previous studies that reported an increase in stem mass with increasing [N] (Balole, 2003; Campiglia et al., 2017).

NUE and KUE decreased with an increase in [N] and [K], respectively (**Figures 4D, F**). The decrease in NUE might be due to an excessive N supply within the tested range, which was evident in growth parameters (number of branches, number of leaves, chlorophyll a and b contents, SD, and SLA) as shown in **Figures 3A, C, E, F** and **4A, C**. This observation aligns with a recent study on cannabis, which shows that higher N input fertilizer treatment decreased NUE (Desaulniers Brousseau et al., 2024). Previous study shows that nutrient use efficiency will decrease with an increase in the application of nutrients when plants exceed their nutrient-holding capacity (Baligar et al., 2001). The slight increase (1%) in PUE with an increase in P application from 30 mg L^-1^ to 100 mg L^-1^ (**Figure 4E**) shows that high P application does not have a significant impact on the growth of cannabis in the vegetative stage. This observation agrees with a previous report, which shows that cannabis plants supplied with 100 mg L^-1^ P performed similarly to those supplied with 30 mg L^-1^ P in the vegetative stage (Shiponi and Bernstein, 2021).

The results for the macronutrient analysis indicated that the optimum nutrient content for the cannabis leaves for TN, P, K, Ca, and S are 0.54, 0.073, 0.27, 0.56, and 0.38 mg g^-1^, respectively. Optimization was not observed in Mg, as its concentration decreased from 0.051 mg g^-1^ to 0.044 mg g^-1^ with increasing [P] and [K]. The interactive effect of [P] and [K] on [Mg] in the cannabis leaves uncovers a complex nutrient interaction for understanding plant-nutrient interaction and has an important practical application for cannabis cultivation. Increased [N] did not affect [Mg] in the cannabis leaves. According to a prior study, cannabis leaves had the highest concentration of macronutrients, followed by the bark and core, except S, which was distributed differently throughout the plant’s organs (Angelini et al., 2014). The reported optimum macronutrient concentrations in this study are slightly higher than a previous study which reported TN, P, K, Ca, and Mg concentrations of 0.28–0.38, 0.03–0.037, 0.18–0.26, 0.20–0.22, 0.086–0.088 mg g^-1^, respectively, in the leaves of cannabis (Wogiatzi et al., 2019). Optimization of P was not observed in any growth parameter but was observed in the leaves of the cannabis plant. This might be an indication that the cannabis plants may not be utilizing P for growth in the vegetative stage but efficiently storing it in the leaves. This finding suggests an additional role of P in cannabis plants, which might be a result of the regulatory or defense mechanism of the plant.

Saloner and Bernstein (2021) reported a decrease in plant growth and inflorescence yield in cannabis plants supplied with N less than 160 mg L^-1^in the vegetative and flowering stages, respectively. A recent study by Desaulniers et al. (2024) shows that low fertilizer treatment (3.2 g N pot^-1^) could provide sizable tetrahydrocannabinol (THC) quantities. Previous report shows that the optimal [N] of medical cannabis was 194 mg L^-1^ (Bevan et al., 2021), 160–320 mg L^-1^ (Saloner and Bernstein, 2021), and 212–261 mg L^-1^ (Caplan et al., 2017a). Cannabis cultivators often supply plants in the flowering stage with relatively high [P] (200 mg L^-1^) on the belief that high P promotes flower development (Bevan et al., 2021). A recent study reported the optimum [P] for cannabis in the flowering stage as 59 mg L^-1^ (Bevan et al., 2021). Increasing [K] in the range of 15–150 mg L^-1^ increased cannabis yield (g/plant) linearly (Yep and Zheng, 2020). Further study shows that cannabis plants supplied with 15 mg L^-1^ K in the vegetative stage had reduced growth, while plants supplied with 60–240 mg L^-1^ K produced substantially more biomass (Saloner et al., 2019). It is reported that some fertilizer companies recommend 300–400 mg L^-1^ K (Bevan et al., 2021).

Balanced nutrition significantly impacts all stages of plant development (Bak and Gaj, 2016). There is a change in the nutritional needs as plants transition from the vegetative to the flowering stage to support bud formation and flower development (Marschner, 2011). In the flowering stage, there is a high demand for P and K as plants focus their energy on producing flowers to ensure high-quality blooms (Mengel and Kirkby, 1987). In medical cannabis, morpho-development during the flowering stage is directly influenced by the size of the plant at the final stages of vegetative growth, which affects the plant’s ability to standardize its secondary metabolites (Saloner and Bernstein, 2020). A study shows that inflorescence yield responded quadratically to N and P, while K was not observed to have a significant effect on inflorescence yield in the tested range of 60–340 mg L^-1^ in the flowering stage of cannabis (Bevan et al., 2021).

We can assess the overall plant health and defense concerning nutrient supply through visual symptoms, yield and growth, root health, and nutrient use efficiency. Assessing plants’ appearance (leaf color, leaf size), measuring H, biomass production, and yield, assessing root morphology and root-to-shoot ratio, and calculating nutrient use efficiency, gene expression, and molecular markers can provide a comprehensive assessment of how nutrients supply impacts plant health and defense mechanisms (Hinsinger, 2001; Marschner, 2011; Fageria, 2012; Taiz et al., 2015).

Traditionally, the energy-intensive Haber-Bosch process for N, sulfuric acid processing for P, and mining ores predominantly from marine deposits for K are used to create inorganic NPK fertilizers (Fageria and Moreira, 2011). A study suggests that economically viable reserves of sulfate and phosphate rocks are being depleted so quickly that they could run out in the next 25–100 years (Kesler, 2007). Runoff from fertilizer solutions with high [N] and [P] in nutrient solution can lead to environmental contamination (Conley et al., 2009; Beerling et al., 2013). According to Schindler et al. (2016), nutrient runoff is the primary cause of the eutrophication of water bodies in many agricultural regions worldwide. Overuse of inorganic fertilizers might disrupt the ecosystem in the soil (Bai et al., 2020), create ocean dead zones (Howard, 2019), and contribute to both air quality reduction and climate warming (Ran et al., 2019). The costs of agricultural fertilizers could fluctuate significantly and become unpredictable due to changes in energy and raw material prices, which would make agricultural sustainability difficult (White and Brown, 2010). Studies show that between September 2021 and 2022, Europe saw a 149% rise in the price of N (European Commission E, 2022). In the second quarter of 2022, Canadian farmers’ fertilizer prices increased by 80.8% when compared to the same period in 2021 (Statistics Canada S, 2022). Fertilizer usage needs to be prudent, and crop production that prioritizes fertilizer management for maximum productivity and sustainability for both commercial and environmental reasons should be the basis for future food security (White and Brown, 2010).

The pH and EC of the nutrient solution significantly affect the plants’ growth. The nutrient solutions in **Supplementary Table S1** showed increased pH and EC. The nitrate/H^+^ cotransporters may be involved in the nitrate uptake, which could be the cause of the increase in pH (Rengel, 2023). According to these studies, high [H^+^] inhibits cation uptake (Bose et al., 2010) and decreases the loading of Mg^2+^, Ca^2+^, Zn^2+^, and Mn^2+^ in the apoplast of root cortical cells, which then reduces their uptake into the symplast (Rengel, 2023). By adjusting the source of the ionic N, namely, the ammonium-to-nitrate ratio in the applied fertilizer, pH can be controlled (Kafkafi and Tarchitzky, 2011; Hawkesford et al., 2023). According to a study by Hawkesford et al. (2012), increasing the ammonium-to-nitrate ratio can cause the pH of the nutrient solution to decrease either through oxidation or through the uptake of ammonium by plant roots. The increase in EC might be due to the modification by plants as they absorb nutrients and water from the nutrient solution, resulting in a simultaneous decrease and increase of some ions in the closed hydroponic system (Trejo-Téllez and Gómez-Merino, 2012). As plants take up water and nutrients, the concentration of the remaining nutrients in the solution can increase, increasing the EC due to a more concentrated solution (Savvas, 2002).

## 5 Conclusion

In the present study, we evaluated the mineral requirement of NPK during the vegetative stage of *C. sativa* using response surface analysis. We found that the optimum concentrations of TN, P, K, Ca, and S in cannabis leaves were 0.54, 0.073, 0.27, 0.56, and 0.38 mg g^-1^, respectively. The cannabis plants might not be utilizing P for growth in the vegetative stage but storing it in the leaves either as a reserve, regulatory mechanism, or defense mechanism. We observed that changes in N concentration did not affect the concentration of Mg in the cannabis leaves; however, an increase in P and K concentrations decreased the concentration of Mg. Additionally, the nutrient interactions of N, P, and K had a significant effect on the vegetative growth parameters of the cannabis plants. Based on the maximum desirability, correlation between the actual and predicted results, and the nutrient use efficiency of this study, we recommend providing cannabis plants in the vegetative stage with nutrient solutions containing 160–200 mg L^-1^ N, 30 mg L^-1^ P, and 60 mg L^-1^ K to achieve the maximum desirable growth parameters. This will help cannabis cultivators significantly by reducing the fertilizer costs and nutrient pollution while achieving the maximum desired growth on cannabis plants. The data obtained from this study is a crucial foundation for understanding the mineral requirements of *C. sativa* in the vegetative stage.

## Data Availability

The original contributions presented in the study are included in the article/**Supplementary Material**. Further inquiries can be directed to the corresponding author.
