# Supplementary material for: Mineral nutrition for Cannabis sativa in the vegetative stage using response surface analysis
Source: Front Plant Sci. 2024 Dec 3;15:1501484. doi: 10.3389/fpls.2024.1501484 (PMC11650207; doi:10.3389/fpls.2024.1501484)
Supplement: Supplementary file 1 [file DataSheet1.docx]

Supplementary Tables

**Table 1.** Electrical conductivity (EC) and pH of initial and final nutrient solutions

| Treatments | Initial EC  (mS cm^-1^) | Final EC  (mS cm^-1^) | Initial pH | Final pH |
| --- | --- | --- | --- | --- |
| 1 | 3118 | 3574 | 5.8 | 6.8 |
| 2 | 2798 | 3290 | 5.8 | 7.1 |
| 3 | 3274 | 3908 | 5.8 | 7.2 |
| 4 | 3150 | 3836 | 5.8 | 6.3 |
| 5 | >4000 | >4000 | 5.8 | 7.1 |
| 6 | 3934 | >4000 | 5.8 | 6.7 |
| 7 | >4000 | >4000 | 5.8 | 6.8 |
| 8 | >4000 | >4000 | 5.8 | 6.0 |
| 9 | 2692 | 3120 | 5.8 | 6.6 |
| 10 | >4000 | >4000 | 5.8 | 6.3 |
| 11 | 3428 | 3822 | 5.8 | 7.2 |
| 12 | 3840 | >4000 | 5.8 | 6.7 |
| 13 | 3710 | >4000 | 5.8 | 7.1 |
| 14 | 3530 | >4000 | 5.8 | 6.5 |
| 15 | 3712 | >4000 | 5.8 | 6.4 |
| 16 | 3709 | >4000 | 5.8 | 6.3 |
| 17 | 3717 | >4000 | 5.8 | 6.7 |
| 18 | 3710 | >4000 | 5.8 | 6.3 |
| 19 | 3712 | >4000 | 5.8 | 6.3 |
| 20 | 3704 | >4000 | 5.8 | 6.0 |

**Table 2.** Statistical analysis of plant growth parameters

| Growth Parameters | R^2^ | R^2^ - Adj | RMSE | F Ratio | Prob > F |
| --- | --- | --- | --- | --- | --- |
| Height | 0.49 | 0.03 | 2.78 | 1.06 | 0.459 |
| Growth index | 0.70 | 0.42 | 25.0 | 2.56 | 0.08 |
| Fresh root mass | 0.81 | 0.64 | 2.24 | 4.82 | 0.0109 |
| Dry root mass | 0.92 | 0.85 | 0.11 | 13.0 | 0.0002 |
| Fresh leaf mass | 0.92 | 0.84 | 0.17 | 12.4 | 0.0003 |
| Dry leaf mass | 0.93 | 0.88 | 0.05 | 15.9 | <.0001 |
| Fresh stem mass | 0.93 | 0.86 | 0.03 | 14.5 | 0.0001 |
| Dry stem mass | 0.94 | 0.88 | 0.004 | 16.0 | <.0001 |
| Stem diameter | 0.79 | 0.59 | 0.06 | 4.10 | 0.02 |
| Number of branches | 0.60 | 0.23 | 1.14 | 1.63 | 0.23 |
| Number of leaves | 0.71 | 0.45 | 6.53 | 2.74 | 0.07 |
| Chlorophyll a content | 0.83 | 0.67 | 0.02 | 5.29 | 0.008 |
| Chlorophyll b content | 0.74 | 0.50 | 0.01 | 3.13 | 0.045 |
| Leaf area | 0.85 | 0.72 | 9.07 | 6.41 | 0.004 |
| Specific leaf area | 0.73 | 0.50 | 0.01 | 3.08 | 0.0047 |
