# Supplementary material for: Mineral nutrition for Cannabis sativa in the vegetative stage using response surface analysis
Source: Front Plant Sci. 2024 Dec 3;15:1501484. doi: 10.3389/fpls.2024.1501484 (PMC11650207; doi:10.3389/fpls.2024.1501484)
Supplement: Supplementary file 2 [file DataSheet2.docx]

Supplementary Material

# Supplementary Figures

| **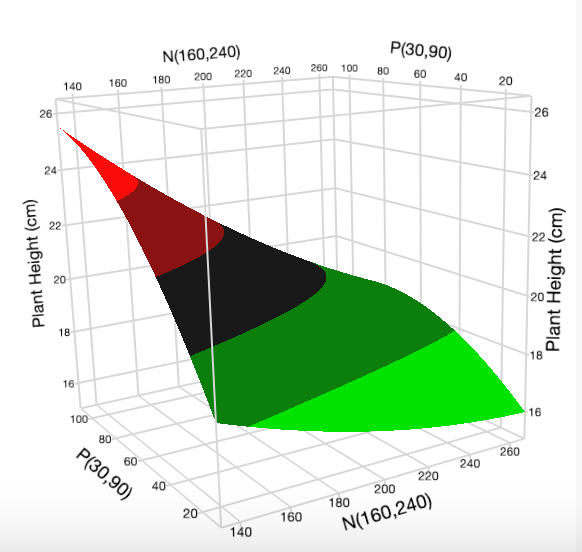Fig. S1A**: Surface plot of N vs. P at K = 123.7 mg L^-1^ for plant height. | **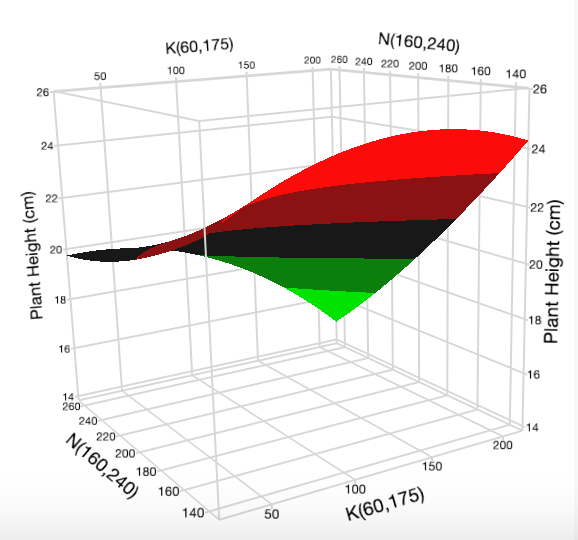Fig. S1B**: Surface plot of K vs. N at P = 90 mg L^-1^ for plant height. | **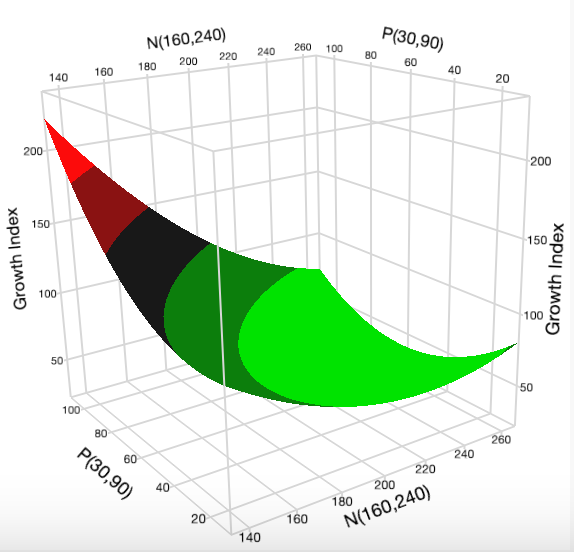Fig. S2A:** Surface plot of N vs. P at K = 175 mg L^-1^ for growth index. |
| --- | --- | --- |
| **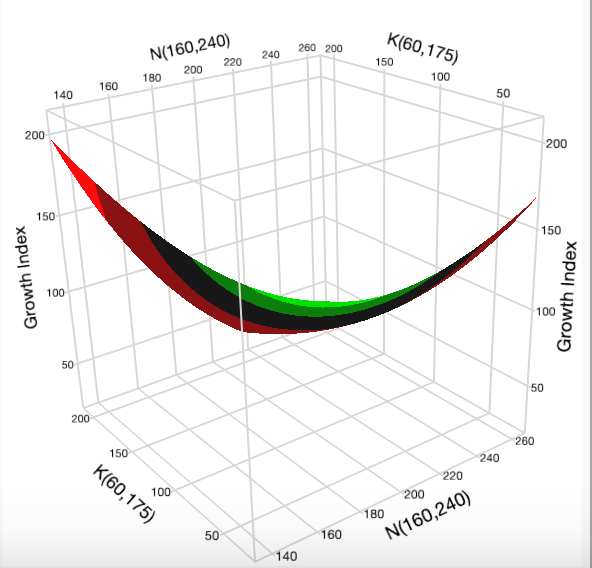Fig. S2B:** Surface plot of N vs. K at P = 90 mg L^-1^ for growth index. | **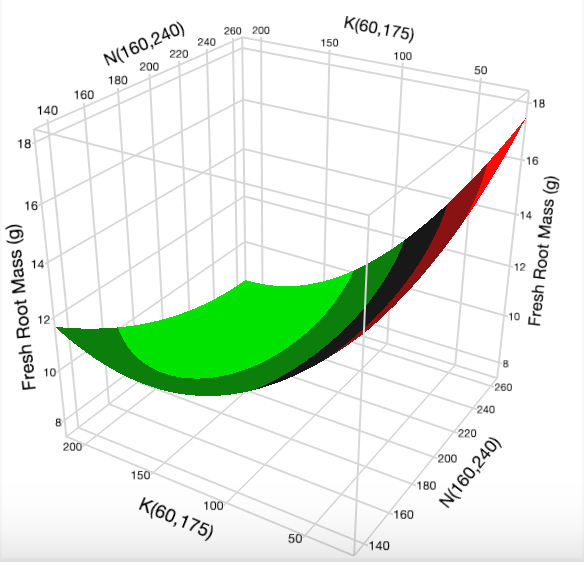Fig. S3A**: Surface plot of N vs. K at P = 90 mg L^-1^ for fresh root mass. | **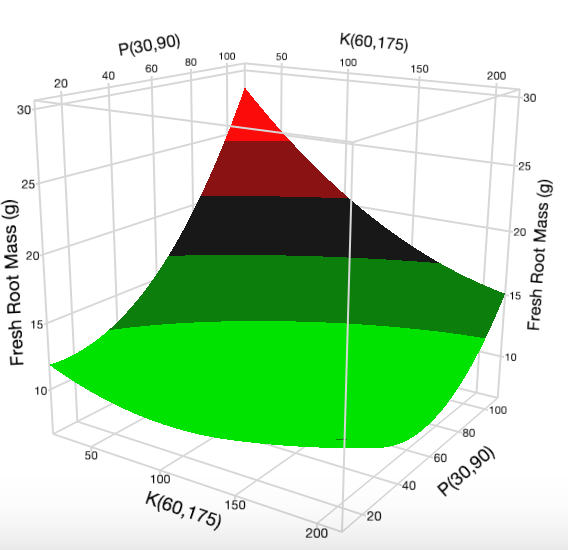Fig. S3B:** Surface plot of P vs. K at N = 240 mg L^-1^ for fresh root mass. |
| 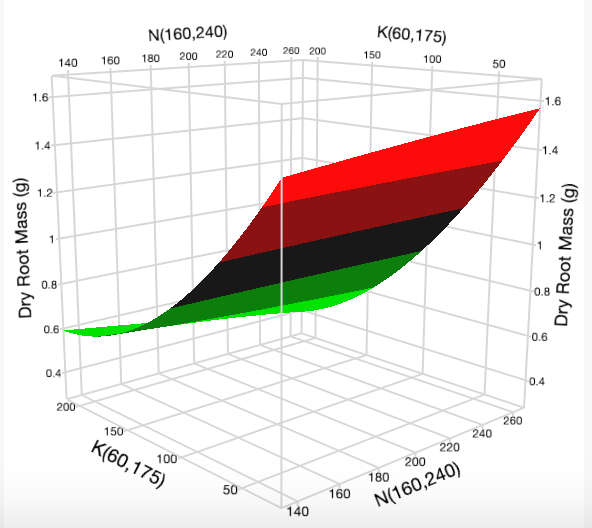  **Fig. S4A:** Surface plot of N vs. K at P = 90 mg L^-1^ for dry root mass. | 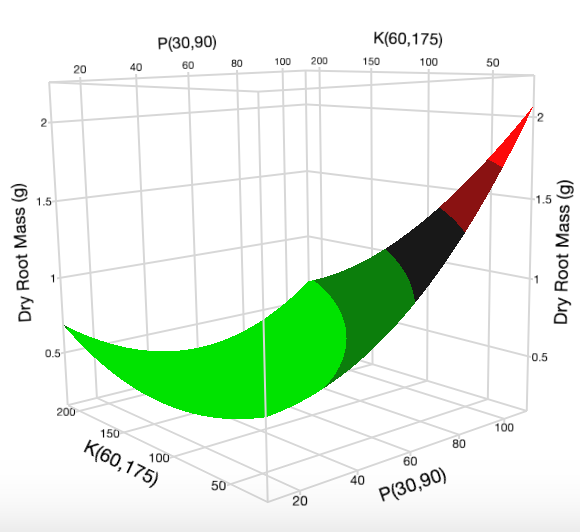  **Fig. S4B:** Surface plot of P vs. K at N = 240 mg L^-1^ for dry root mass. | 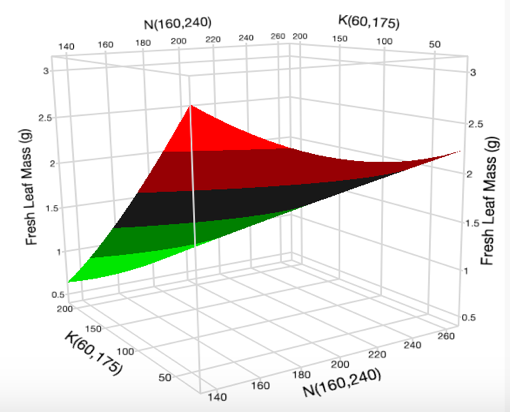  **Fig. S5A:** Surface plot of N vs. K at P = 90 mg L^-1^ for fresh leaf mass. |
| 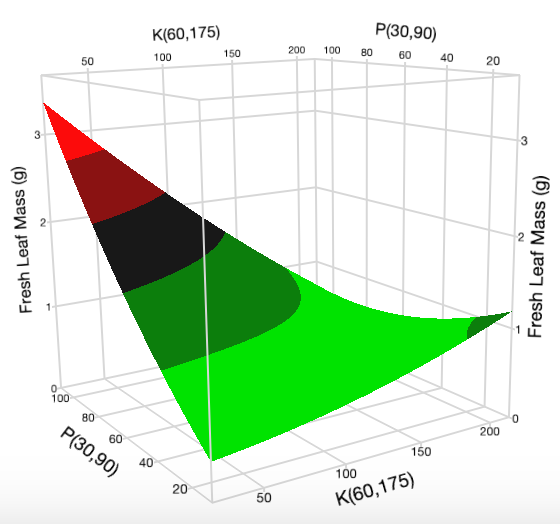**Fig. S5B**: Surface plot of K vs. P at N = 160 mg L^-1^ for fresh leaf mass. | 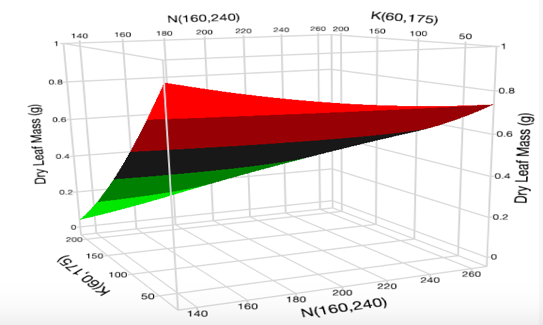**Fig. S6A**: Surface plot of N vs. K at P = 90 mg L^-1^ for dry leaf mass. | 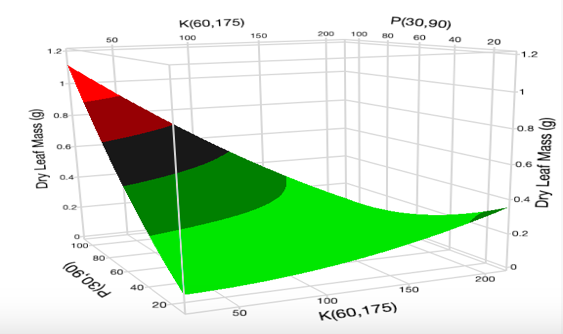**Fig. S6B:** Surface plot of K vs. P at N = 160 mg L^-1^ for dry leaf mass. |
| 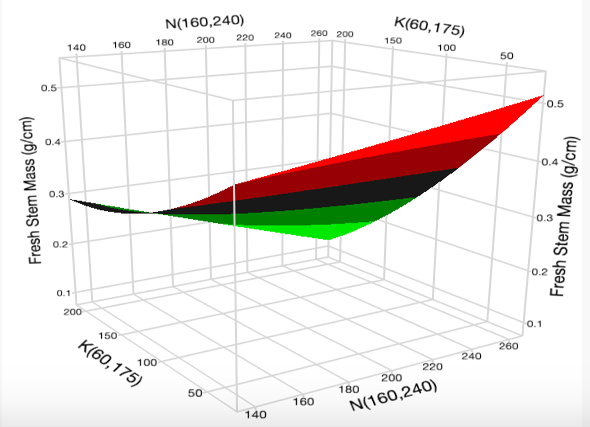**Fig. S7A**: Surface plot of N vs. K at P = 90 mg L^-1^ for fresh stem mass. | 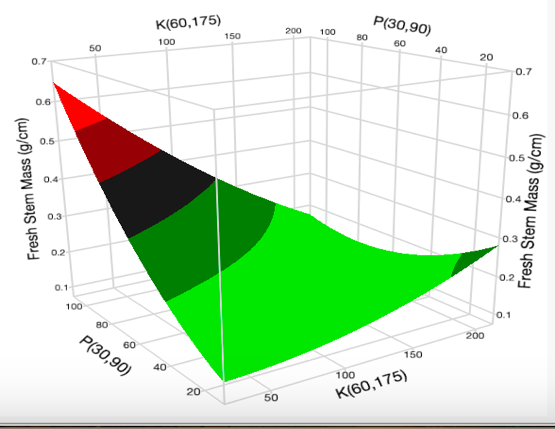**Fig. S7B:** Surface plot of K vs. P at N = 160 mg L^-1^ for fresh stem mass. | 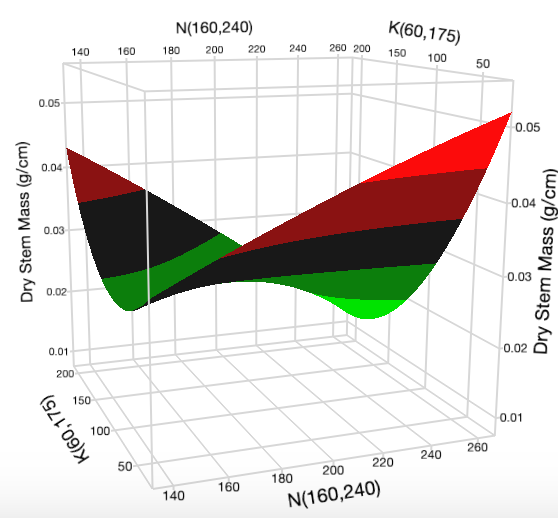**Fig. S8A:** Surface plot of N vs. K at P = 90 mg L^-1^ for dry stem mass. |
| 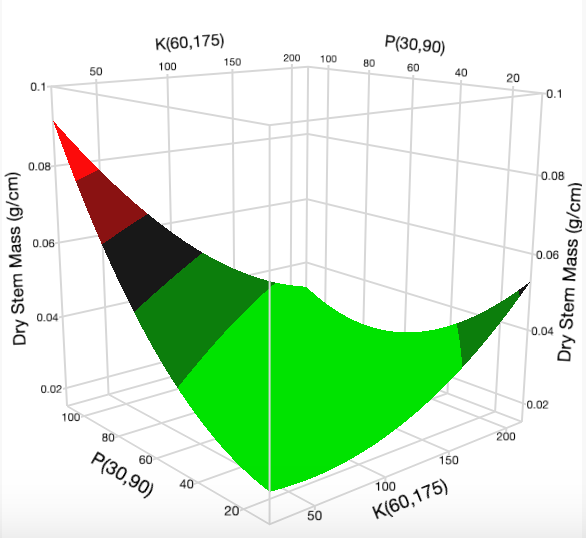  **Fig. S8B**: Surface plot of K vs. P at N = 160 mg L^-1^ for dry stem mass. | 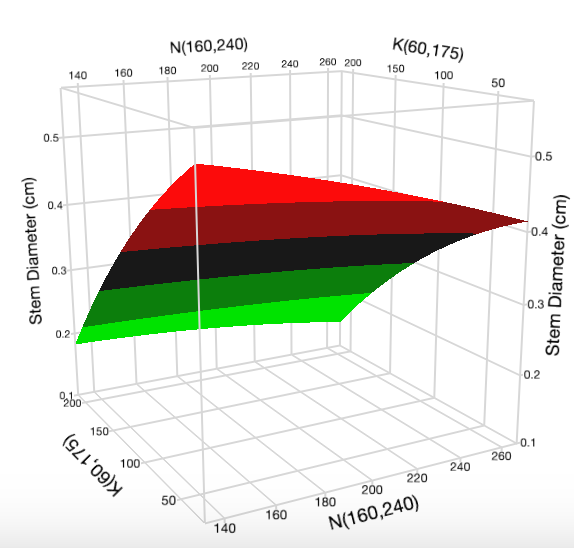**Fig. S9A:** Surface plot of N vs. K at P = 90 mg L^-1^ for stem diameter. | 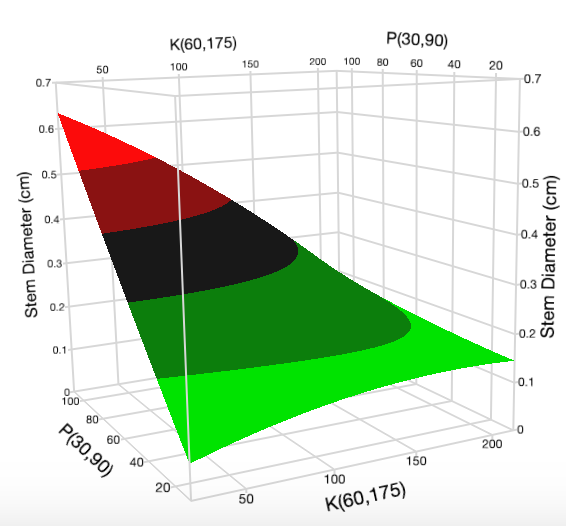**Fig. S9B:** Surface plot of K vs. P at N = 160 mg L^-1^ for stem diameter. |
| 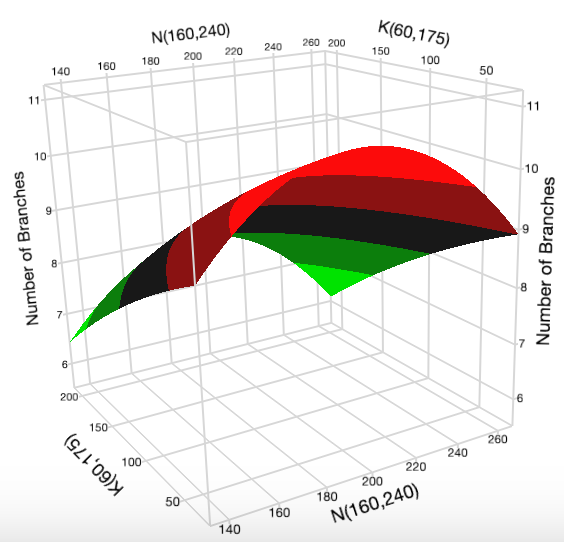**Fig. S10A**: Surface plot of N vs. K at P = 90 mg L^-1^ for number of branches. | 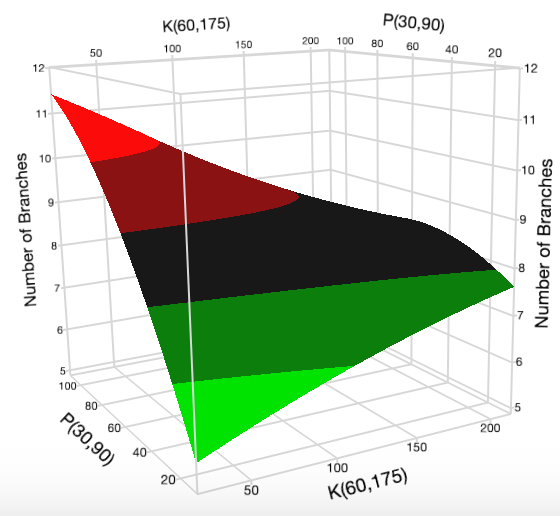**Fig. S10B**: Surface plot of K vs. P at N = 199.4 mg L^-1^ for number of branches. | 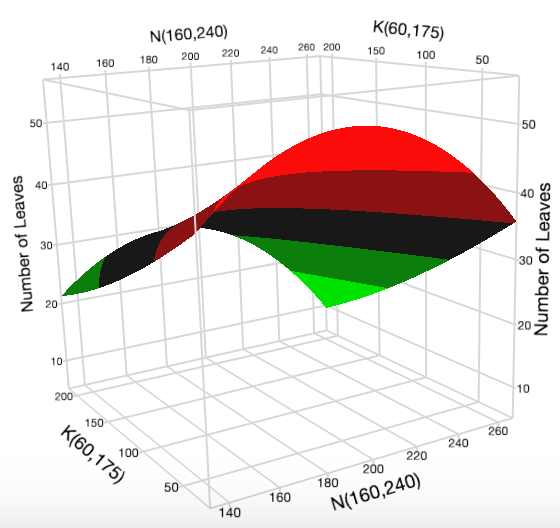  **Fig. S11A:** Surface plot of N vs. K at P = 90 mg L^-1^ for number of leaves. |
| 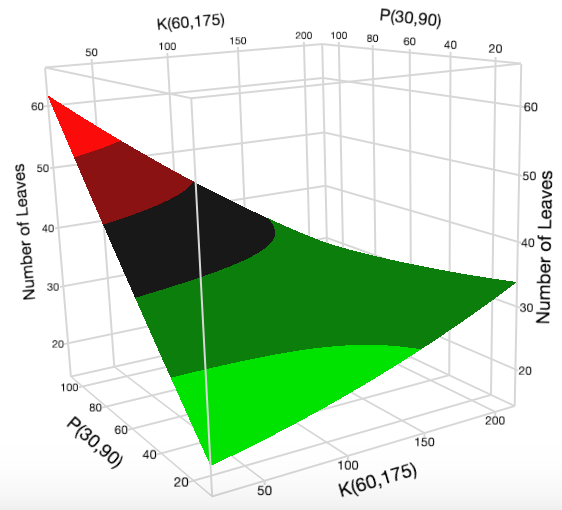**Fig. S11B:** Surface plot of K vs. P at N = 90 mg L^-1^ for number of leaves | 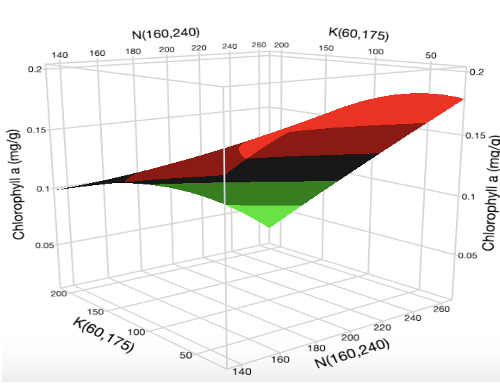 **Fig. S12A:** Surface plot of N vs. K at P = 90 mg L^-1^ for chlorophyll a content. | 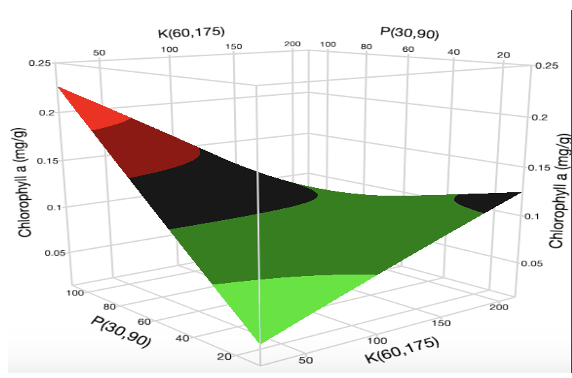  **Fig. S12B:** Surface plot of K vs. P at N = 203.6 mg L^-1^ for chlorophyll a content. |
| 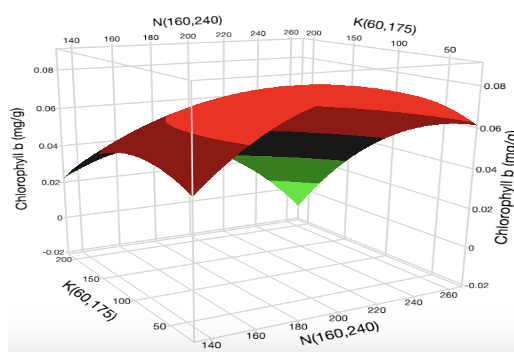**Fig. S13A**: Surface plot of N vs. K at P = 90 mg L^-1^ for chlorophyll b content. | 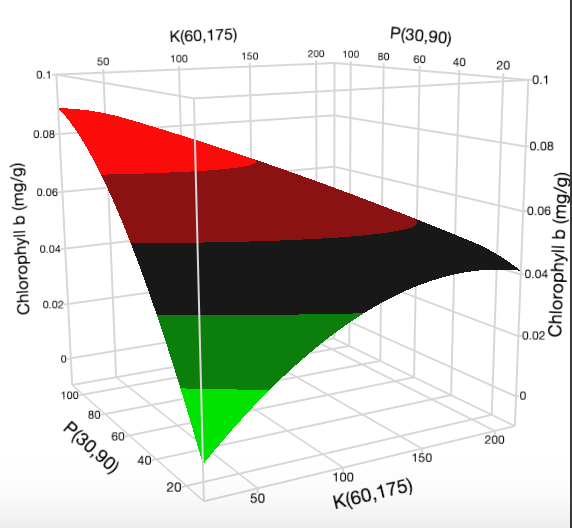**Fig. S13B:** Surface plot of K vs. P at N = 200 mg L^-1^ for chlorophyll b content. | 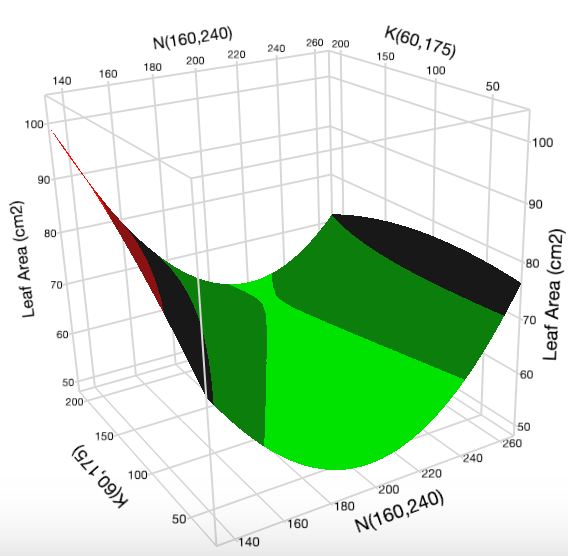**Fig. S14A**: Surface plot of N vs. K at P = 90 mg L^-1^ for leaf area. |
| 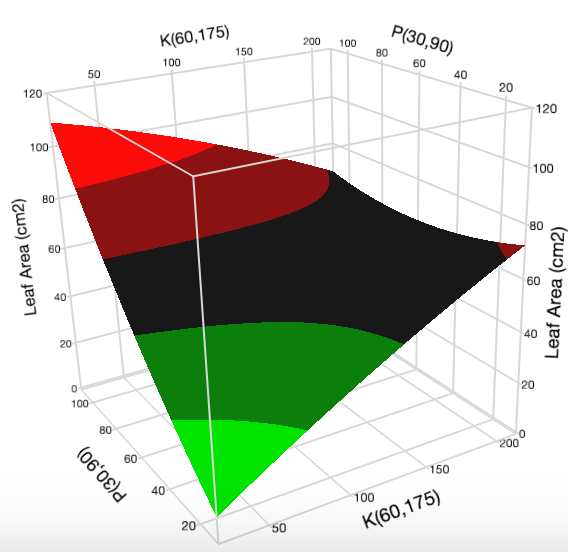  **Fig. S14B:** Surface plot of K vs. P at N = 160 mg L^-1^ for leaf area. | **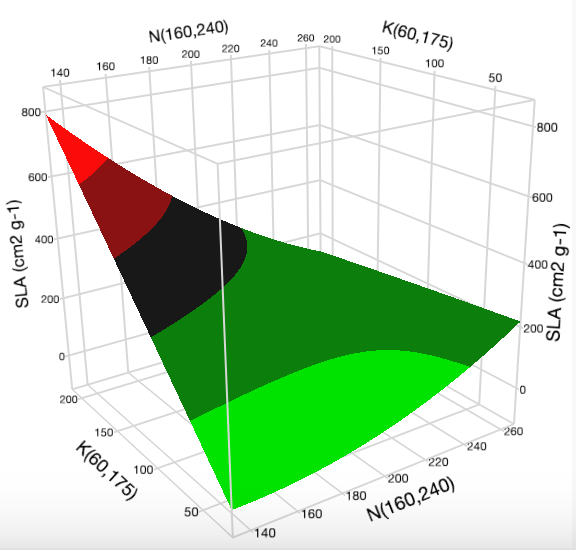**  **Fig. S15A:** Surface plot of N vs. K at P = 90 mg L^-1^ for specific leaf area. | **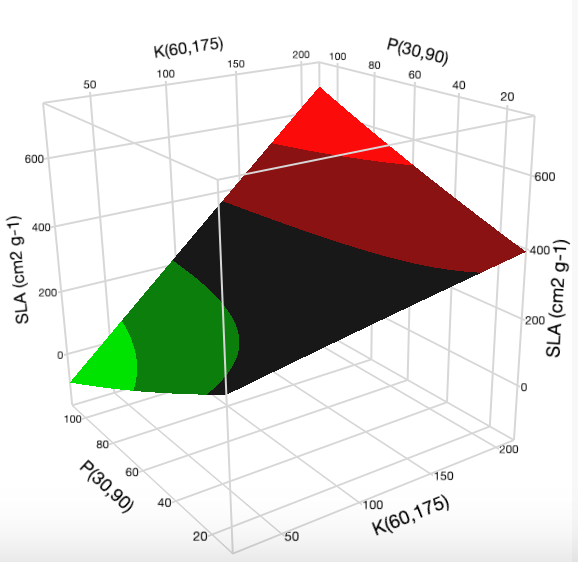**  **Fig. S15B:** Surface plot of K vs. P at N = 160 mg L^-1^ for specific leaf area. |
| 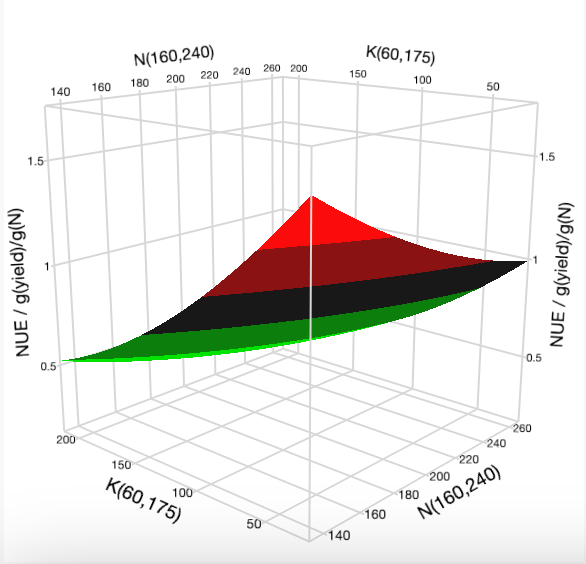**Fig. S16A**: Surface plot of N vs. K at P = 90 mg L^-1^ for nitrogen use efficiency | 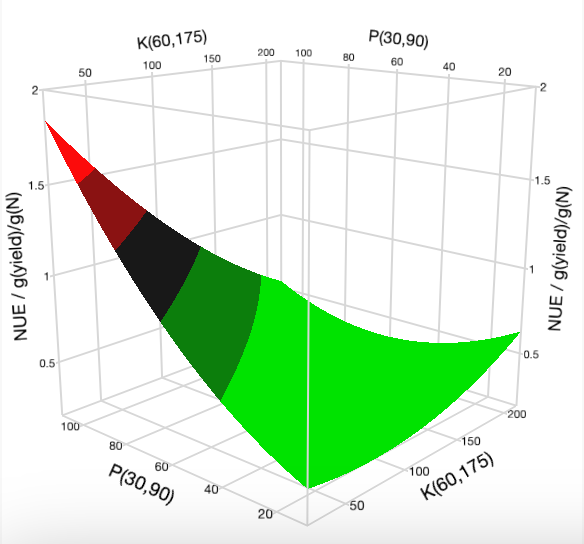**Fig. S16B:** Surface plot of K vs. P at N = 160 mg L^-1^ for nitrogen use efficiency. | 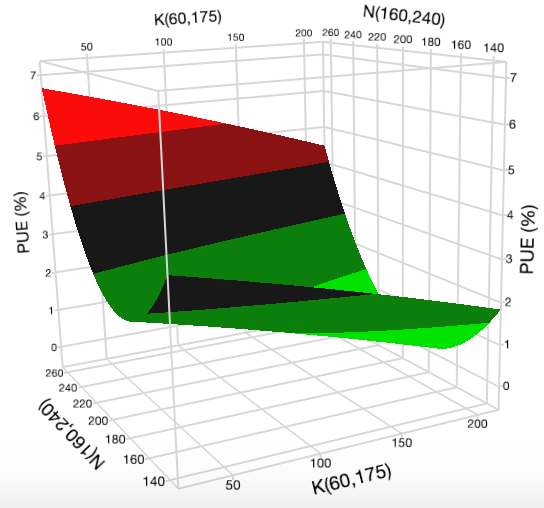**Fig. S17A:** Surface plot of K vs. N at P = 90 mg L^-1^ for phosphorus use efficiency. |
| 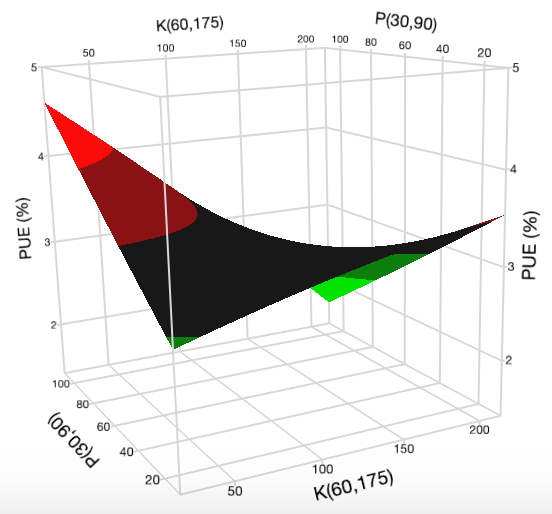  **Fig. S17B:** Surface plot of K vs. P at N = 200 mg L^-1^ for phosphorus use efficiency. | 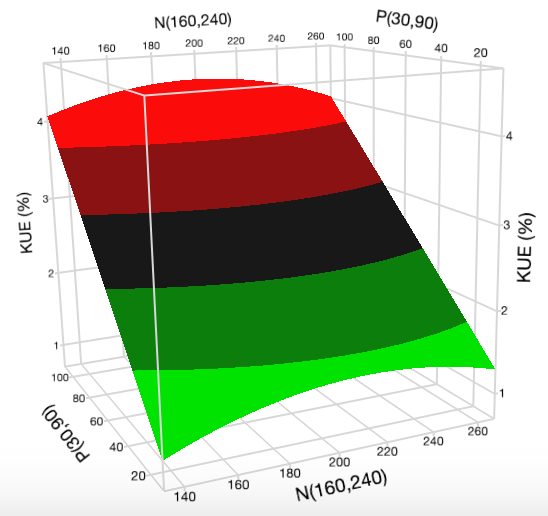  **Fig. S18A:** Surface plot of N vs. P at K = 117.5 mg L^-1^ for potassium use efficiency. | 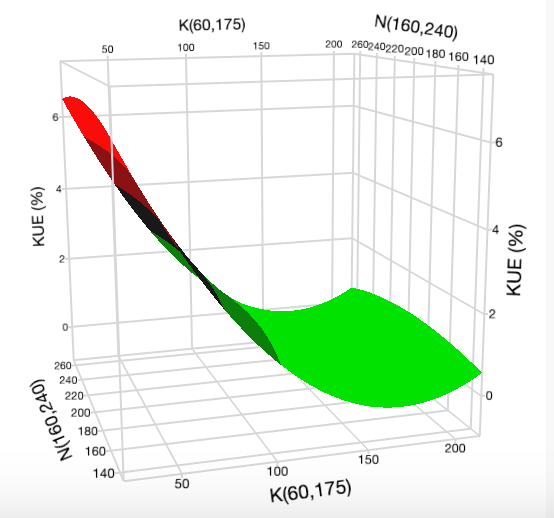  **Fig. S18B:** Surface plot of K vs. N at P = 90 mg L^-1^ for potassium use efficiency. |
| 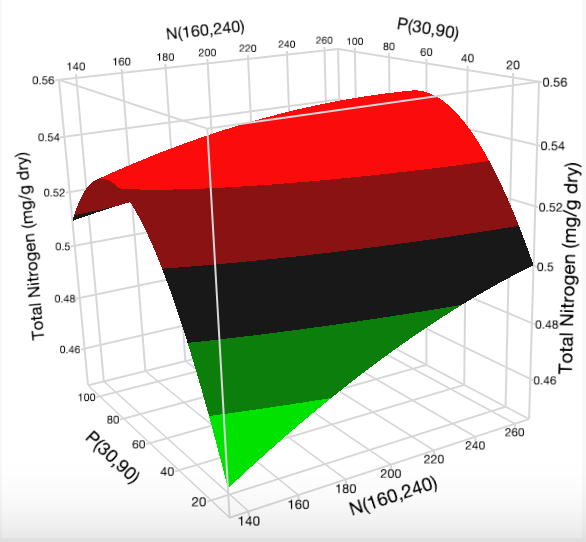  **Fig. 19A:** Surface plot of N vs P at K = 117.5 mg L^-1^ for total nitrogen content in dry cannabis leaf. | 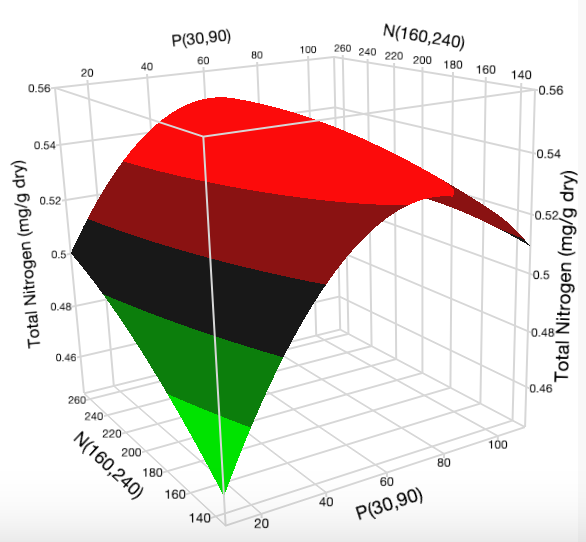  **Fig 19B:** Surface plot of P vs N at K = 117.5 mg L^-1^ for total nitrogen content in dry cannabis leaf. | 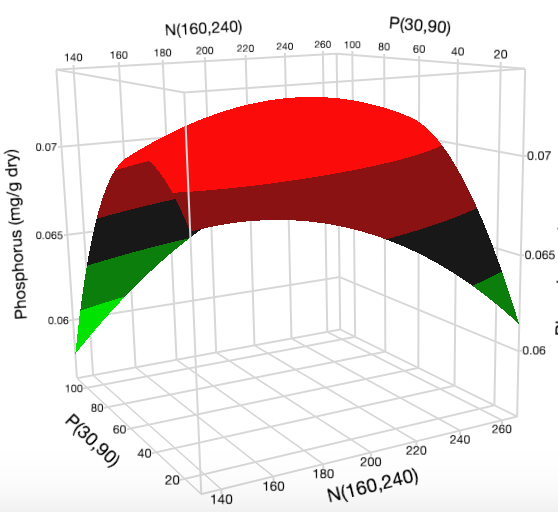  **Fig. 20A:** Surface plot of N vs P at K = 117.5 mg L^-1^ for phosphorus content in dry cannabis leaf. |
| 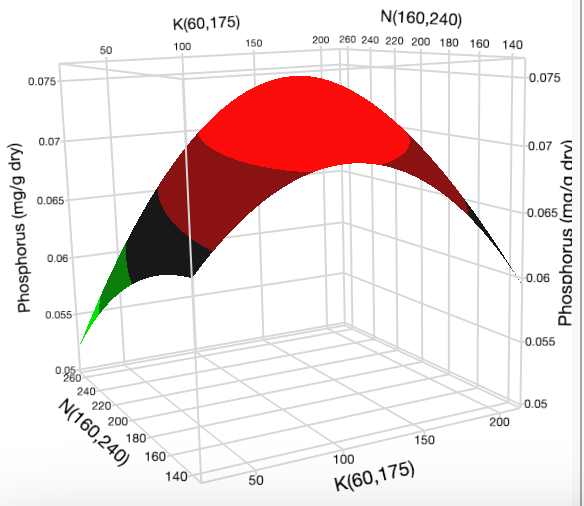  **Fig. 20B:** Surface of plot K vs N at P = 60 mg L^-1^ for phosphorus content in dry cannabis leaf. | 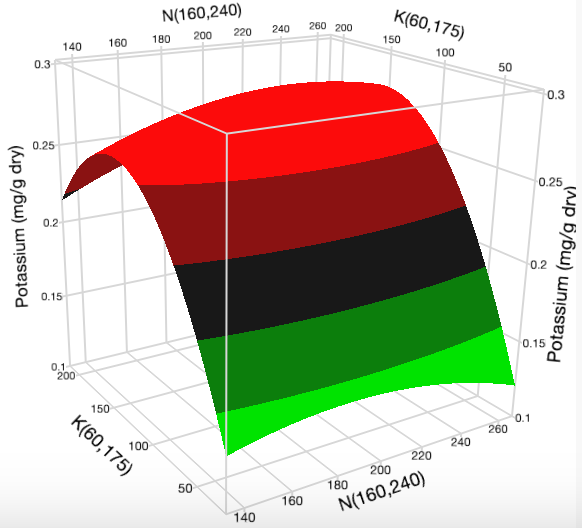  **Fig. 21A**: Surface plot of N vs K at P = 60 mg L^-1^ for potassium content in dry cannabis leaf. | 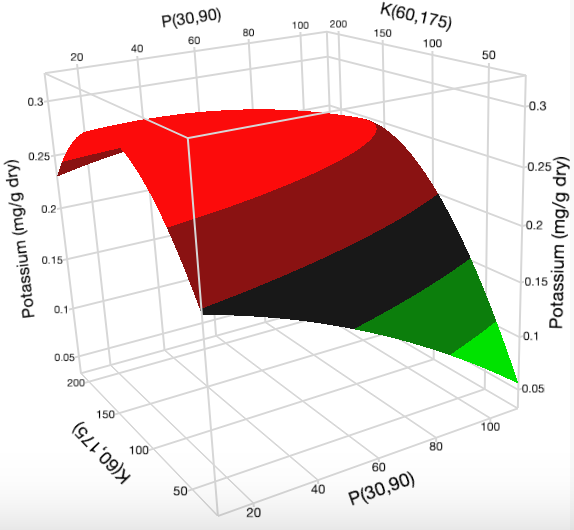  **Fig. 21B:** Surface plot of P vs K at N = 200 mg L^-1^ for potassium content in dry cannabis leaf. |
| **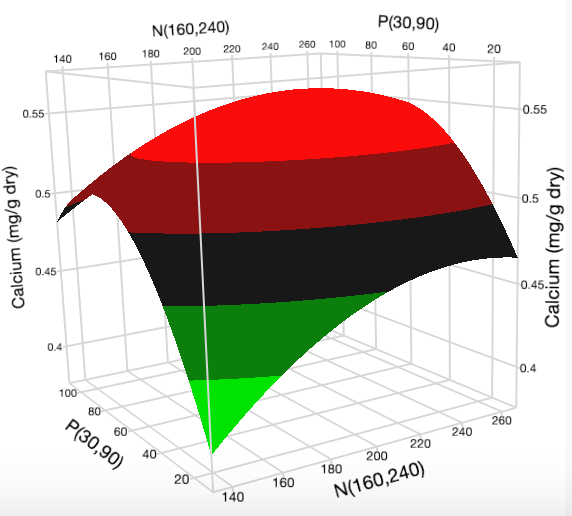**  **Fig. 22A**: Surface plot of N vs P at K = 117.5 mg L^-1^ for calcium content in dry cannabis leaf. | **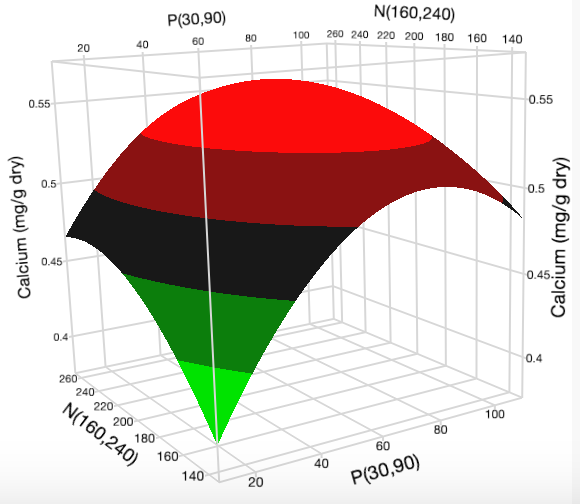**  **Fig. 22B:** Surface plot of P vs N at K = 117.5 mg L^-1^ for calcium content in dry cannabis leaf. | **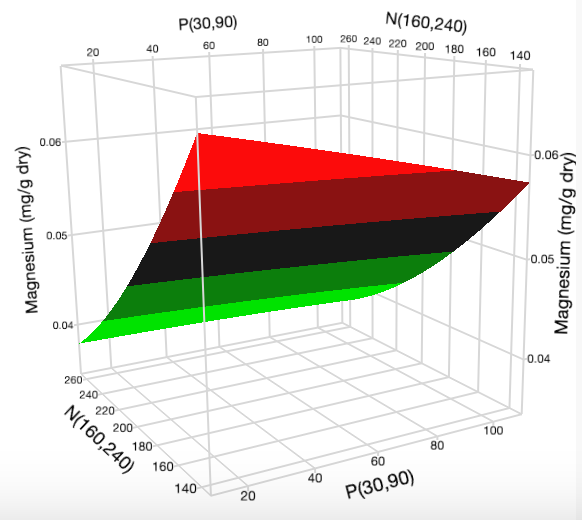**  **Fig. 23A**: Surface plot of P vs N at K = 117.5 mg L^-1^ for magnesium content in dry cannabis leaf. |
| **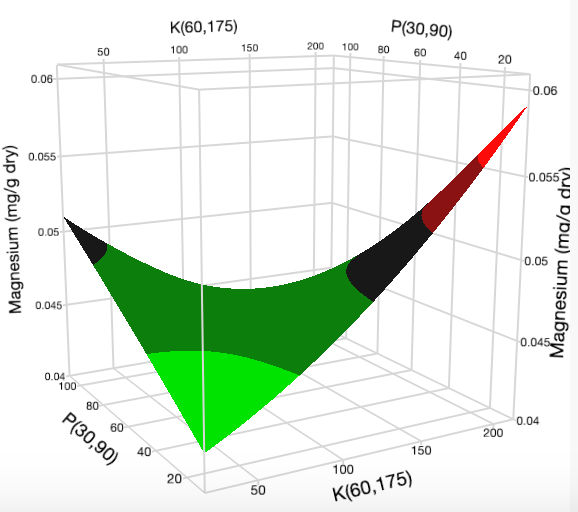**  **Fig. 23B:** Surface plot of K vs P at N = 200 mg L^-1^ for magnesium content in dry cannabis leaf. | **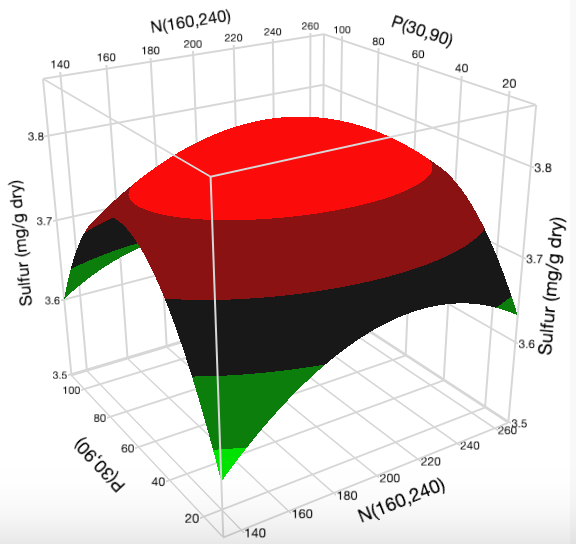**  **Fig. 24A:** Surface plot of N vs P at K = 117.5 mg L^-1^ for sulfur content in dry cannabis leaf. | **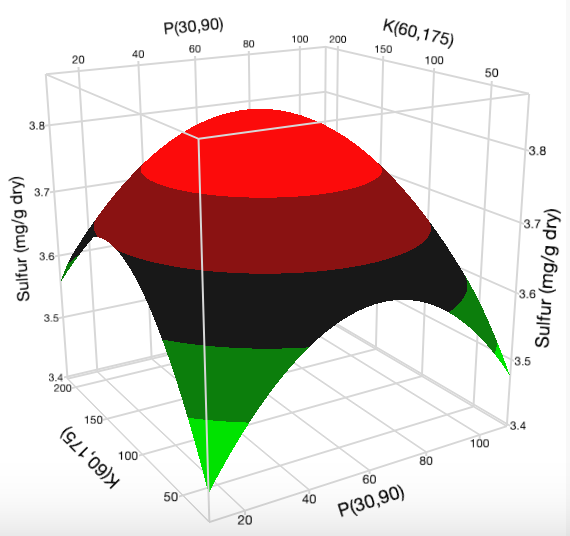**  **Fig. 24B**: Surface plot of P vs K at N = 200 for sulfur content in dry cannabis leaf. |

|  |  |  |
| --- | --- | --- |
|  |  |  |

**Figure 23.** Plots comparing the actual and predicted RSM values from RSM for (A) plant height, (B) growth index, (C) fresh root mass, (D) dry root mass (E) fresh leaf mass, and (F) dry leaf mass for the various nutrient treatments.

|  |  |  |
| --- | --- | --- |
|  |  |  |

**Figure 24.** Plots comparing the actual and predicted RSM values from RSM for (A) fresh stem mass, (B) dry stem mass, (C) stem diameter, (D) number of branches, (E) number of leaves, and (F) chlorophyll a content for the various nutrient treatments.

| **** | **** |
| --- | --- |
| **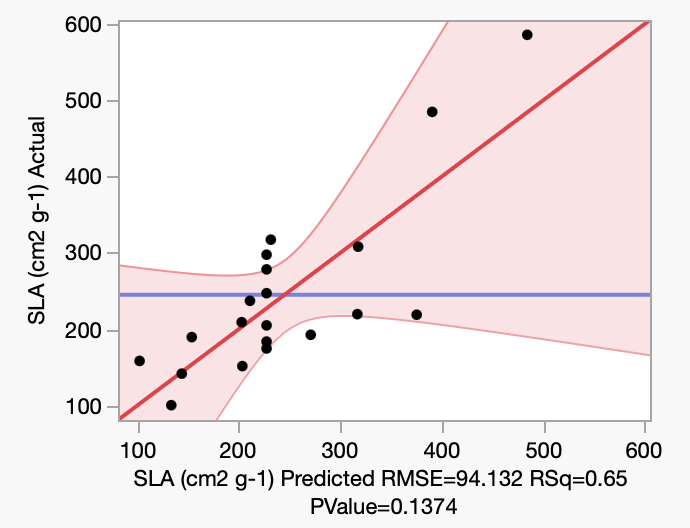** | **** |

**Figure 25.** Plots comparing the actual and predicted RSM values from RSM for (A) chlorophyll b content, (B) leaf area, and (C) specific leaf area for the various nutrient treatments
